# A New Catalog of Structural Variants in 1,301 *A. thaliana* Lines from Africa, Eurasia, and North America Reveals a Signature of Balancing Selection at Defense Response Genes

**DOI:** 10.1093/molbev/msaa309

**Published:** 2020-11-28

**Authors:** Mehmet Göktay, Andrea Fulgione, Angela M Hancock

**Affiliations:** Max Planck Institute for Plant Breeding Research, Cologne, Germany

**Keywords:** presence–absence variation, core genome, dispensable genome, balancing selection, R-genes, F-box

## Abstract

Genomic variation in the model plant *Arabidopsis thaliana* has been extensively used to understand evolutionary processes in natural populations, mainly focusing on single-nucleotide polymorphisms. Conversely, structural variation has been largely ignored in spite of its potential to dramatically affect phenotype. Here, we identify 155,440 indels and structural variants ranging in size from 1 bp to 10 kb, including presence/absence variants (PAVs), inversions, and tandem duplications in 1,301 *A. thaliana* natural accessions from Morocco, Madeira, Europe, Asia, and North America. We show evidence for strong purifying selection on PAVs in genes, in particular for housekeeping genes and homeobox genes, and we find that PAVs are concentrated in defense-related genes (R-genes, secondary metabolites) and F-box genes. This implies the presence of a “core” genome underlying basic cellular processes and a “flexible” genome that includes genes that may be important in spatially or temporally varying selection. Further, we find an excess of intermediate frequency PAVs in defense response genes in nearly all populations studied, consistent with a history of balancing selection on this class of genes. Finally, we find that PAVs in genes involved in the cold requirement for flowering (vernalization) and drought response are strongly associated with temperature at the sites of origin.

## Introduction

The model plant *Arabidopsis thaliana* has been important for deciphering core physiological, developmental, and adaptive processes (Somerville and Koornneef 2002; Provart et al. 2016). *Arabidopsis thaliana* grows naturally in diverse environments throughout Eurasia and Africa, where populations are exposed to differing selective pressures. Natural variation of *A. thaliana* has mainly been studied using single-nucleotide polymorphisms (SNPs) (Alonso-Blanco et al. 2016; Durvasula et al. 2017; Zou et al. 2017; Fulgione et al. 2018). However, SNPs represent only a subset of the variation in the genome. Structural variants (SVs) are generally defined as genomic variants larger than 50 bp including presence/absence variants (PAVs), tandem duplications, inversions, translocations, and complex SVs (Eisfeldt et al. 2019; Kosugi et al. 2019). Compared with SNPs, SVs are likely to cause more dramatic effects on gene functions and phenotypes (Kosugi et al. 2019). Structural variation can also have indirect effects through repression of meiotic crossovers (Sturtevant 1926; Morgan et al. 2017; Rowan et al. 2019).

SVs are common in humans (estimated at >20K variants per individual) and only a subset has been associated with phenotypes, including disease (Weischenfeldt et al. 2013; Abel et al. 2020; Ho et al. 2020). For example, in humans, an approximately 3-Mb deletion on chromosome 15 (chr15q11-13—paternal) was shown to result in loss of function of multiple genes and cause Prader–Willi syndrome (Weischenfeldt et al. 2013), and Charcot–Marie–Tooth disease is known to be caused by a duplication event on Chromosome 17 (chr17p12), which damages peripheral nerves (Weischenfeldt et al. 2013). In Drosophila, several individual SVs are associated with fitness and display clinal patterns (González et al. 2010; Kapun et al. 2016; Durmaz et al. 2018).

Plant genomes seem to be exceptional at tolerating structural variation. Extensive variation in gene content has been observed across individuals in rice (Wang et al. 2018; Fuentes et al. 2019; Choi et al. 2020), maize (Springer et al. 2009; Sun et al. 2018), *A. thaliana* (Cao et al. 2011; Zmienko et al. 2020), and grapes (Zhou et al. 2019) and several studies have implicated individual SVs in trait variation. Structural variation resulting from transposable element insertions has been shown to play roles in domestication in maize (Studer et al. 2011) and in gene expression divergence between *Arabidopsis* species (Hollister et al. 2011) and copy number variation is linked with several postdomestication traits (Lye and Purugganan 2019). Other studies identified large-scale chromosomal inversions associated with salt tolerance in Mimulus (Lowry and Willis 2010), flowering time in *A. thaliana* (Fransz et al. 2016), berry color in grapes (Zhou et al. 2019), awn length in basmati rice (Choi et al. 2020), and the loss of the jointed fruit pedicel (Soyk et al. 2019) and fruit size (Alonge et al. 2020) in tomato.

In this study, we identify SVs in 1,301 *A. thaliana* accessions from Africa, Europe, Asia, and North America and examine the evolutionary history of these SV polymorphisms. First, we assess the precision and sensitivity of several available methods by comparison to simulations and long-read data to produce an analysis pipeline. Given the limitations of short-read data, we focus our analyses on indels and SVs (including PAVs, tandem duplications, and inversions) smaller than 10 kb. We identify structural variation in *A. thaliana* natural accessions examine their global patterns of polymorphism. We identify sets of “core” highly conserved genes and variable “dispensable” genes, a subset of which show evidence for balancing selection. Finally, we examine evidence of local adaptation on PAVs based on correlations with environmental variables. This analysis reveals strong correlations for PAVs in three genes involved in the vernalization (cold) response for flowering as well as genes involved in drought and heat tolerance. We make our variant calls available together with information about the level of support for each call, which can be used for flexible integration of SVs into existing analysis pipelines.

## Results

### Performance Comparison of SV Callers

Identifying and calling SVs in short-read data is not a trivial task. To identify the best software for calling SVs, we compared the performance of three popular tools: PINDEL, DELLY, and LUMPYEXPRESS (Ye et al. 2009; Rausch et al. 2012; Layer et al. 2014). We conducted comparisons to SVs produced in simulations as well as to calls made from long-read Pacific Biosciences (hereafter PacBio) data that we generated.

**Fig. 1.**
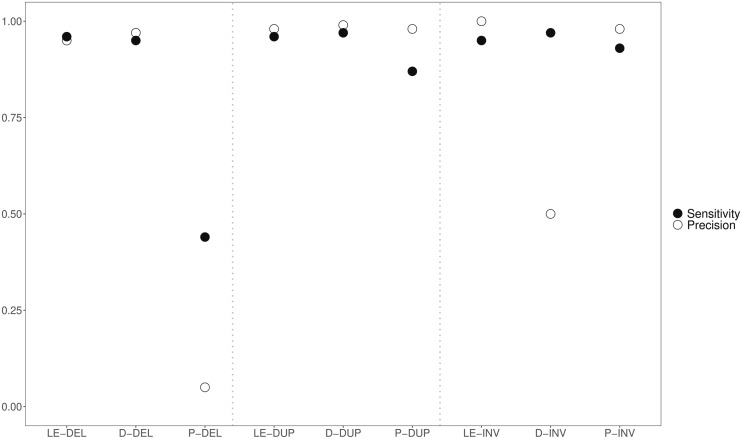
Comparison of SV callers based on simulations and PacBio. Abbreviations along the *x* axis represent the combination of method used and variant type and are defined as follows: LE, LumpyExpress; D, Delly; P, Pindel; DEL, deletion; DUP, duplication; INV, inversion.

For the simulation-based approach, LUMPYEXPRESS outperformed DELLY and PINDEL (fig. 1). Both precision and sensitivity for LUMPYEXPRESS and DELLY were very high (≥95%) for deletions and tandem duplications, whereas both measures were much lower for PINDEL (44% and 5%). For inversions, LUMPYEXPRESS had much higher precision (100%) than DELLY (50%), which called many false positives and PINDEL performed slightly worse than LUMPYEXPRESS. In silico simulations provide information about the performance of SV detection tools under ideal conditions, but they assume simple scenarios. However, in real population-level data, SVs may be more complex, with multiple SVs and mutations occurring at the same locus. To explore this, we also compared the performance of the three tools relative to calls from long-read data (PacBio) in an empirical case. For this, we used the Cvi-0 accession, which is one of the very most diverged accessions compared with the Col-0 (TAIR10) reference and therefore represents a particularly challenging case, where complex structural variation is likely. We note that this case is challenging both for long-read and short-read callers.

We considered the results from long-read calls (PacBio) to represent a “high confidence” set and then assessed how well calls from short-read data using LUMPYEXPRESS, DELLY, and PINDEL agreed with this high confidence set. Although PacBio data are much more reliable for large SVs, error rates in PacBio data are high for individual SNPs and short INDELs. Therefore, in an effort to maximize the true positive rate for calls with the PacBio data, we only considered SVs between 50 bp and 10 kb in length in this comparative analysis. Supplementary figure S1, Supplementary Material online, shows the performance of LUMPYEXPRESS, DELLY, and PINDEL on short-read data from Cvi-0 relative to the high PacBio-based confidence set. For the tandem duplications and inversions, agreement was never above 45%. However, deletions and associated PAVs were called with relatively high agreement. LUMPYEXPRESS called 62% of the high confidence set of PAVs with 80% agreement to SNIFFLES and DELLY called 64% of PAVs with 74% agreement to SNIFFLES.

Given the discrepancy between the very high sensitivity and precision, we observed in simulations compared with the lower levels with PacBio data, we were interested in better understanding what drives the concordant and discordant calls in short- and long-read data. We focused on the LUMPYEXPRESS software and examined alignments in randomly chosen regions where calls based on PacBio data (using SNIFFLES) and Illumina data (using LUMPYEXPRESS) agreed and where they differed. Supplementary figures S2–S4, Supplementary Material online, show five randomly selected regions of agreement between calls with Illumina and PacBio data, five randomly selected PAVs identified from Illumina data but not from PacBio data and five randomly selected PAVs identified in PacBio data but not in Illumina data. Somewhat surprisingly, we found that the discrepancies tended to result from false negatives in one or the other technology and/or offsets in the true breakpoints. Based on this sample, the errors appeared to be equally prevalent in long- and short-read data. Discrepancies tended occur in regions where alignments are imperfect so that called PAVs are likely true positives that went undetected by either the short-read or long-read calling approaches. The discrepant regions tended to be complex (possibly involving multiple layered SVs) and/or repetitive regions, which are known to be problematic for calling both SNPs and SVs in long- and short-read data. Overall, the lower matching to PacBio long-read data appears to result from the general problem that calling SVs in complex genomic regions is equally error-prone using short- (Illumina) and long-read (PacBio) data. Based on the results of comparisons to both simulations and real data, we decided to use LUMPYEXPRESS for identifying and calling SVs in the global sample set.

### Alignment, SV Calling, and Genotyping in 1,301 Diverse *A. thaliana* Accessions

**Fig. 2.**
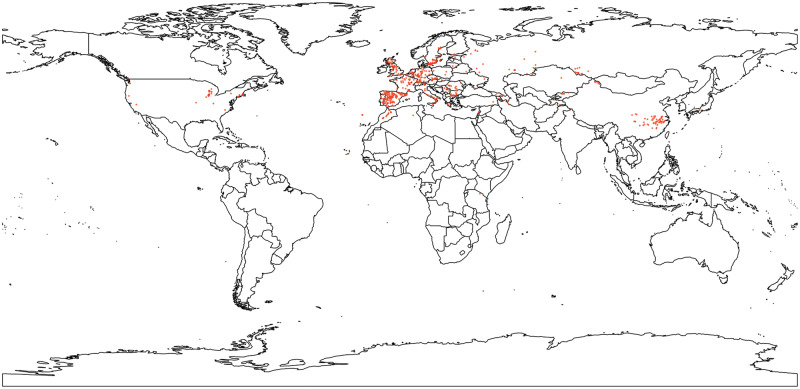
Geographical distribution of *Arabidopsis thaliana* samples included in this study.

Next, we examined the patterns of variation in structural polymorphisms across diverse wild *A. thaliana* accessions. Figure 2 shows the geographic distribution of the samples that we included in this study. We identified 155,440 SVs ranging in size from 1 bp to 10 kb among 1,301 accessions using a pipeline consisting of LUMPYEXPRESS, SVTYPER, and SVTOOLS (supplementary fig. S5, Supplementary Material online). This set includes 124,905 PAVs, 25,061 tandem duplications, and 5,474 inversions. Although we make the entire set of SVs publicly available (PRJEB38975), our subsequent analyses focus on PAV polymorphisms, which were called with the greatest precision in our testing data set. For researchers interested in using this data set, we make information available about the strength of evidence for SVs based on the number of reads supporting each SV as well as the type of evidence (split read and/or discordant reads), which could be used for further (i.e., more stringent) filtering.

### PAVs Recapitulate Population Clustering Obtained from SNPs

**Fig. 3.**
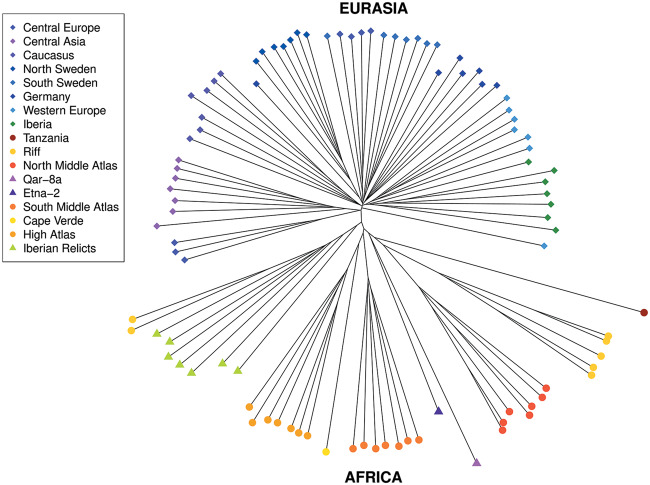
Neighbor-joining tree including seven representative samples for each population. To produce the neighbor-joining tree, the full set of accessions was down-sampled to achieve similar numbers for different geographic regions. The tree is based on 95 accessions which contain a total of 9,062 PAVs. The tree shows a clear separation between Eurasia and Africa, which further validates PAV calls. PAVs give reliable information to recapitulate previous findings from SNP data.

We examined the global pattern of polymorphism in our total set of PAVs in order to assess whether these variants recapitulated the signals found in SNP data. We consider this to be a further test to validate the PAV genotyping because if the quality of PAV genotypes is high, we expect to see global patterns that are similar to those found using SNP data. We clustered a representative subset of diverse accessions using PAVs and compared this with results obtained previously from SNP data (Durvasula et al. 2017). We found that the overall structure of the NJ-tree based on PAVs (fig. 3) recapitulates that based on SNPs (figure 2A in Durvasula et al. 2017). First, both SNPs and PAVs clearly separate the Eurasian nonrelict clade (comprising the majority of Eurasian accessions) from the relict clades (highly diverged groups mainly found in the Iberian Peninsula and Africa). Second, similar to the pattern observed for SNPs, the Eurasian clade has a nearly star-shaped pattern with little reticulation whereas the relict clades are more deeply reticulated with longer internal branches. Finally, clustering using SVs recovered the fine-scale differentiation of population clusters within Africa and most of that within Eurasia.

### Identification of Genomic Hotspots of PAVs and Conserved Regions

Although reduction in effective population size due to selfing is expected to reduce the efficiency of selection at weakly deleterious alleles (Schemske and Lande 1985; Charlesworth et al. 1990; Charlesworth and Wright 2001), variants that are lethal or have severe effects in the homozygous state are more often exposed to selection in selfing compared with outcrossing species (Stebbins 1950; Shields 1982; Schemske and Lande 1985; Glémin 2007). To determine whether there is evidence for purifying selection acting to limit PAVs, we examined their frequencies and genome-wide distributions*.* At a broad scale, PAVs were nearly uniformly distributed across chromosomes, except in pericentromeric regions where they were more common (supplementary fig. S6, Supplementary Material online). When we focused specifically on genic and nongenic regions within chromosome arms, we found that PAVs were enriched by 3.5-fold in nongenic relative to genic regions (χ^2^ = 1.8E7, df = 1, *P* = 1E-16), consistent with stronger purifying selection in genic regions (Charlesworth et al. 1993) (table 1). Purifying selection on weakly deleterious alleles is expected to increase the relative proportion of low-frequency variants in functional relative to neutrally evolving genomic regions (Nielsen 2005). We compared the unfolded site frequency spectra (SFS) (supplementary fig. S7, Supplementary Material online) for genic and intergenic loci for all populations where ancestral state could be assigned based on consensus calls in a set of divergent genomes. We did not find enrichment of low-frequency variants in genic regions relative to nongenic regions; rather, we found a slight deficit in all cases. Taken together our results suggest that new genic PAVs often have very strong deleterious effects and are quickly removed by purifying selection, so that they are not found in segregating variation. Conversely, the set of genic PAVs that are segregating at appreciable frequencies do not show evidence of purifying selection relative to intragenic PAVs.

**Table 1.** Distribution of PAVs across the Genome for 1,301 *Arabidopsis thaliana* Accessions.

Genomic Partition	Mb	Number of PAVs	Mean Length	Median Length	Mb PAVs	Proportion Containing PAVs
Whole genome	119,146,348	124,905	681.8	91	39,696,386	0.333
Intergenic	59,041,854	87,627	547.9	87	30,766,220	0.521
Genic	60,104,494	37,278	996.1	100	8,920,166	0.148

Next, we asked which genes or pathways showed deficits of PAVs. To do that, we extracted all genes that never contained any PAVs in any samples and performed gene ontology (GO) enrichment analysis. We tested significance using both Fisher’s exact tests (FET) and a more conservative permutation-based approach that corrects for clustering of signals in the genome (Gowinda) (Huang et al. 2009; Kofler et al. 2012). Categories that were significantly enriched with both FET and Gowinda include translation (Benjamini-corrected *P* values for FET = 5.4E-33 and for Gowinda = 9.13E-05) and translational elongation (Benjamini *P* values for FET = 1.1E-4 and for Gowinda = 2.93E-02). These categories include tRNAs and rRNAs, which are classical examples of housekeeping genes (Rifkind et al. 1976). In addition, the GO term regulation of DNA-templated transcription, which contains homeobox genes and transcription factors was also strongly enriched (Benjamini *P* value for FET = 6.5E-03 and Gowinda = 9.12E-05). Homeobox genes play an important role in body plan specification of higher organisms during early stages of embryogenesis (Duverger and Morasso 2008). Additional GO term categories that were found to be enriched either in FET or Gowinda analyses are listed in (supplementary tables S2 and S3, Supplementary Material online).

Although genes show a deficit of PAVs relative to nongenic regions overall, some types of genes may be more likely to contain PAVs than others. To identify these, we extracted locations of all genes that overlap at least one PAV among the 1,301 samples and performed GO enrichment analysis using both FET and the Gowinda permutation-based approach (Huang et al. 2009; Kofler et al. 2012). The results are reported in (table 2).

**Table 2.** Multiple-Test Significant GO Terms for Genes Carrying PAV Polymorphisms Among the Total Set of 1,301 Samples.

GO Term	Enrichment Score	Benjamini (Corrected *P* Value)	Test Statistics
Signal transduction	1.2	2.2E-6	One-tailed Fisher’s exact test
Defense response	1.2	1.4E-3	One-tailed Fisher’s exact test
SCF-dependent proteasomal ubiquitin-dependent protein catabolic process	1.4	1.4E-3	One-tailed Fisher’s exact test
Cell–cell signaling	2.4	1.84E-3	Permutation-based test
Lipid transport	1.6	1.84E-3	Permutation-based test
Lipid localization	1.6	1.84E-3	Permutation-based test
Lignan metabolic process	2.6	4.46E-3	Permutation-based test
Lignan biosynthetic process	2.6	4.46E-3	Permutation-based test

Note.—Two methods (FET and Gowinda) were employed to be able to identify enriched categories with different test statistics.

Signal transduction was the most significantly enriched class with FET, and the enrichment was driven largely by defense-related genes mainly consisting of TIR-NBS, TIR-NBS-LRR, LLR, and TIR classes and secondary metabolites. Other enriched classes included the more specific defense response category and a category related to the production of Lignans, a class of secondary metabolites (Bagniewska-Zadworna et al. 2014). In addition, SCF-dependent proteasomal ubiquitin-dependent protein catabolic processes were enriched, which contains many F-box genes. F-box genes have previously been noted to evolve rapidly (Xu et al. 2009) and are known to be involved in several crucial processes related to environmental stress response including embryogenesis, hormonal responses, seedling development, floral organogenesis, senescence, and pathogen resistance (Xu et al. 2009). Based on Gowinda analysis, the most enriched GO terms that we identified are known to be related to stress including cell–cell signaling (Xing and Laroche 2011), lipid transport, and localization (Yeats and Rose 2008). The defense response GO term was also enriched in marginal tests using Gowinda (*P* = 2E-2), but the enrichment was not significant with Benjamini correction. This discrepancy is likely due to the extreme clustering of defense genes across the genome.

Next, we examined the patterns within populations. We extracted all PAVs for each population and performed GO term enrichment. Table 3 shows defense response enrichment from FET and Gowinda. The population-based analysis reveals significant Benjamini enrichment of defense response genes with PAVs across almost all populations for both enrichment tests. The signal was somewhat weaker in Central Europe, Germany, and Iberian nonrelicts, where defense response genes were not enriched at the Benjamini significance level using the Gowinda method. Other categories that were often enriched across individual populations are signal transduction and secondary metabolite biosynthetic processes (supplementary tables S4–S19, Supplementary Material online).

**Table 3.** Defense Response Enrichment with Significance Assessed by One-Tailed Fisher’s Exact Tests and Gowinda for Each Population.

Population	One-Tailed Fisher’s Exact Test		Gowinda	
	Enrichment Score	Benjamini Corrected *P* Value	Enrichment Score	Benjamini Corrected *P* Value
Asia	1.4	1.20E-07	1.2	1.83E-03
Central Europe	1.3	2.60E-05	1.1	1.17E-01
Germany	1.4	1.60E-08	1.1	3.38E-01
High Atlas (Morocco)	1.6	9.40E-11	1.4	2.45E-03
Iberian Relicts	1.5	4.20E-10	1.3	2.23E-03
Iberian nonrelicts	1.4	8.30E-10	1.1	7.94E-02
Italy, Balkans, and Caucasus	1.4	2.70E-09	1.2	1.25E-03
North Middle Atlas (Morocco)	1.7	2.00E-13	1.5	5.70-03
North Sweden	1.6	2.30E-13	1.4	5.43E-03
Riff (Morocco)	1.7	8.80E-11	1.4	2.83E-03
South Middle Atlas (Morocco)	1.6	1.60E-12	1.4	2.77E-03
South Sweden	1.4	1.70E-09	1.3	1.55E-03
Western Europe	1.4	1.50E-11	1.2	8.62E-03
Madeira	1.7	2.00E-09	1.4	5.72E-03
Yangtze River Basin (PopY)	1.6	5.50E-12	1.5	6.03E-03
North-Western China (PopN)	1.6	2.40E-08	1.5	4.06E-03

**Fig. 4.**
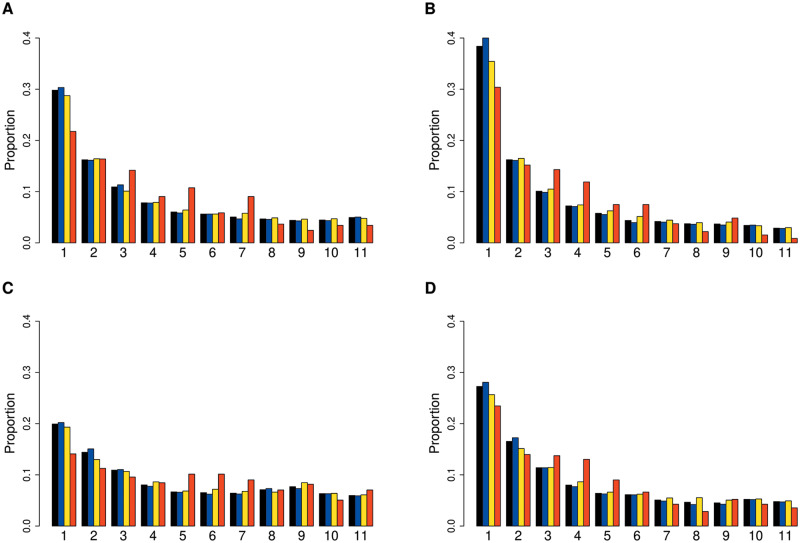
SFS of PAVs for whole genome (black), intergenic (blue), genic(yellow), and defense genes (red) in four representative populations: (*A*) South Middle Atlas, (*B*) Iberian Relicts, (*C*) Asia (PopN), and (*D*) North Sweden.

**Fig. 5.**
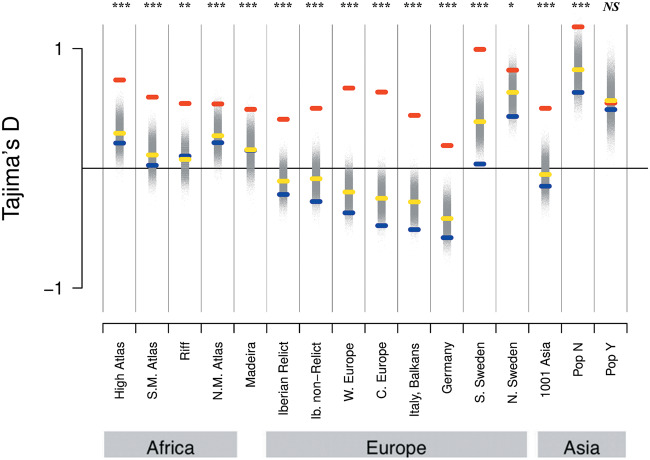
Tajima’s *D* of intergenic (blue), genic (yellow), and defense (red) loci. To assess significance for Tajima’s *D* in defense response genes, 100K sets of genic PAVs were randomly subsampled to match the number of defense response PAVs and these distributions are shown in gray. Asterisks denote level of significance; *P < 0.5 × 10^−2^, **P < 5 × 10^−3^; ***P < 5 × 10^−4^; and *NS*, not significant.

Since we observed very strong signals in defense response genes, we were interested in better understanding the evolutionary forces acting on these. We examined the SFS of defense-related genes relative to the complete set of genic and nongenic categories for each population. In most populations, defense PAV frequencies were shifted towards intermediate levels relative to other genes (fig. 4 and supplementary fig. S8, Supplementary Material online). To quantitatively compare the frequency distribution of PAVs overlapping defense genes with the total sets of genes and intergenic regions, we used the Tajima’s *D* statistic. This statistic summarizes the information in the SFS such that an excess of intermediate frequency variants results in a more positive Tajima’s *D*, and an excess of low-frequency variants results in a more negative Tajima’s *D* (Tajima 1989). For each population, Tajima’s *D* was more positive for defense genes compared with the total set of genes. To assess significance for this result, we randomly subsampled genic PAVs to match the number of defense response PAVs 100K times and calculated an empirical *P* value based on the distribution of Tajima’s *D* in the sampled data sets. Population bottlenecks and expansions affect the frequency spectrum and therefore the genome-wide value of Tajima’s *D*. We found that Tajima’s *D* was highly significantly elevated in defense PAVs in all populations except Yangtze River Basin (PopY) (not significant; *P* = 0.551) and Northern Sweden, where the significance was marginal (*P* = 0.0254) (fig. 5 and supplementary table S20, Supplementary Material online). Both populations are known to have experienced strong bottlenecks in the past (Huber et al. 2014; Zou et al. 2017) and accordingly have a higher Tajima’s *D* in intergenic regions. We further assessed evidence of long-term balancing selection at defense loci based on a signal of long-range LD among SNPs in these regions. For this, we used the BetaScan method (Siewert and Voight 2017, 2020). We calculated beta scores for each population and tested for enrichment of the defense genes in the 5% tail of the distribution of beta scores. For each population, we found a significant enrichment of these defense genes in the tail of this distribution (supplementary table S21, Supplementary Material online). Taken together, the shift towards intermediate frequency variation resulting in a shift to more positive Tajima’s *D* and the observed enrichment of long-range LD in defense genes is consistent with balancing selection on this class of loci.

### Correlation with Environmental Variables (GWAS)

**Fig. 6.**
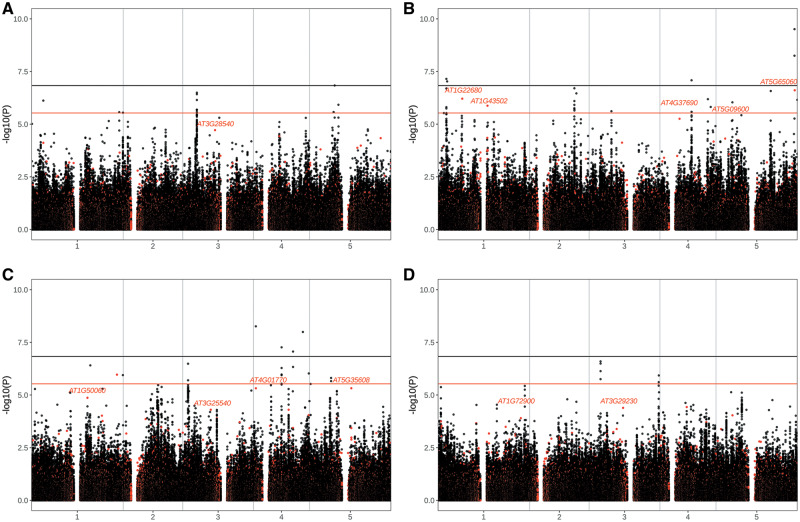
Plots of −log_10_(*P* values) from genome-wide association mapping between PAVs and SNPs with environmental variables, including (*A*) Bio5: Maximum temperature of the warmest month, (*B*) Bio 6: Minimum temperature of the coldest month, (*C*) Bio13: Precipitation of the wettest month, and (*D*) Bio14: Precipitation of the driest month. Red and black dots indicate PAVs and SNPs, respectively. Horizontal lines show genome-wide significance thresholds with Bonferroni correction for SNPs (black) and PAVs (red).

We next asked whether there was evidence for involvement of specific PAVs in local adaptation based on associations with environmental variables. For this, we accessed data for four representative environmental variables (Bio5: Maximum temperature of the warmest month, Bio6: Minimum temperature of the coldest month, Bio13: Precipitation of the wettest month and Bio14: Precipitation of the driest month) from the WorldClim database (Fick and Hijmans 2017). To identify SVs that were correlated with the environment while controlling for potential confounding effects of population structure, we performed GWAS with a linear mixed model that controls for relatedness using a kinship matrix as a random variable (Zhou and Stephens 2012) and environmental variables as phenotypes (fig. 6*A*–*D*). We also conducted the same analyses for SNPs so that we could compare the signals between the two classes of variants.

Several genic PAVs (PAVs within 10 kb of genes) strongly associated with minimum temperature in the coldest month (Bio6) were related to the timing of flowering and photosynthesis (fig. 6*B*). There were strong correlations for multiple PAVs in a *MADS AFFECTING FLOWERING* (*MAF*) gene cluster at the bottom of chromosome 5, including a Bonferroni significant association with a PAV that impacts *MAF3* as well as several less significantly correlated (*P* value range: 2 × 10^−7^ to 1 × 10^−2^) PAVs that affect other genes in this cluster. *MAF3* and other MAF cluster genes work together with *FLM* to repress expression of *FT* and inhibit flowering and play a role in flowering in response to cold (vernalization) (Ratcliffe et al. 2003; Gu et al. 2013). Photosynthetic efficiency is reduced in cold, which can result in damage from build-up of reactive oxygen species (ROS) (Tripathy and Oelmüller 2012; Prinzenberg et al. 2020). Several PAVs in genes involved in photosynthesis and ROS production were among the most strongly correlated with minimum temperature, including *AT1G22700* (*tetratricopeptide repeat [TPR]-like superfamily protein*) (*P* value: 4.8 × 10^−7^), *AT1G22710* (*SUC2*) (*P* value: 4.8 × 10^−7^), *AT1G43560* (*THIOREDOXIN Y2*) (*P* value: 1.3 × 10^−6^), *AT5G09600* (*Succinate dehydrogenase 3-1*) (*P* value: 3.5 × 10^−5^), and *AT5G22140* (FAD/NAD(P)-binding oxidoreductase family protein) (*P* value: 3.6 × 10^−5^).

For maximum temperature in the warmest month (Bio5) PAVs at three genes involved in photomorphogenesis (hypocotyl elongation), a phenotype with a plastic temperature-mediated effect, were among the strongest correlations (fig. 6*A*). These included AT5G11260 (*ELONGATED HYPOCOTYL 5*) (*P* value: 1.5 × 10^−5^), AT5G42350 (CFK1) (*P* value: 8.3 × 10^−5^), and AT5G58140 (*PHOTOTROPIN 2*) (*P* value: 2.4 × 10^−5^). Among the strongest correlations with precipitation in the wettest month (Bio13) were PAVs within 10 kb of several genes and gene clusters involved in cell wall morphogenesis: *RGXT1* (AT4G01770) and *RGXT2* (AT4G01750) (*P* value: 4.9 × 10^−6^), *Walls Are Thin 1* (AT1G75500) (*P* value: 1 × 10^−6^), and *EXPA18* (AT1G62980) and *KNAT7* (AT1G62990) (*P* value: 9.5 × 10^−5^) (fig. 6*C*). A PAV in a cluster of several immune response genes (AT1G72890–AT1G72950) was among the most correlated with precipitation in the driest month (Bio14) (*P* value: 1.9 × 10^−5^) (fig. 6*D*).

Although having information about PAVs can be specifically useful to identify candidates in GWAS for functional follow-up analysis and therefore creates added value compared with SNPs alone, some information may be partially redundant with SNP results in the sense that LD between PAVs and SNPs will result in statistical associations with both types of variants. To examine this, we compared the signals we found with PAVs to those with SNPs in the same regions. Overall, there are many fewer PAVs than SNPs in the genome (124905 vs. 8033502). The average distance between two any SNPs is 14.4 bp and the average distance between PAVs is 771 bp, and the average distance from any PAV to the nearest SNP is 332 bp so that LD between any two SNPs tends to be higher than LD between any two PAVs. To determine the extent to which we identified novel variation by including PAVs in our analysis, we examined how well a SNP represents each strongly correlated PAV. To this end, for each PAV with a GWAS *P* value <0.001, we identified the SNP with the highest LD (*r*^2^) within a 10-kb window. The mean and SD of *r*^2^ between a PAV with this significance level and the most correlated SNP for each of the four traits were as follows: Bio5: mean = 0.70, SD = 0.25; Bio6: mean = 0.75, SD = 0.24; Bio13: mean = 0.70, SD = 0.23; and Bio14: mean = 0.76, SD = 0.25. The full distributions of these most correlated SNPs are shown in supplementary figure S11, Supplementary Material online. The set of PAVs we identified may be useful specifically to reveal missing heritability in GWAS and for identifying causative variants (when these turn out to be SVs) even in cases where they are tagged by SNPs.

## Discussion

In this study, we identified SVs among 1,301 diverse *A. thaliana* accessions using publicly available NGS data and we make the entire set available, which includes 124,905 PAVs, 25,061 tandem duplications, and 5,474 inversions. For researchers interested in using these data, we note that the data set includes information about the strength of evidence for SVs based on the number of reads supporting each SV as well as the type of evidence (split read and/or discordant reads), which could be used for further filtering. Here, we focused our population genetic analyses on PAVs because these were called with the highest accuracy based on our analyses.

Given that the genic PAVs we identify are expected to often disrupt gene function, our results were consistent with the idea that a genome is made up of an essential “core” set of genes as well as a “flexible” set of genes, which may be dispensable depending on the specific local selection pressures faced by an organism. In our analyses, we found evidence for purifying selection based on the low occurrence of PAVs in genic compared with nongenic regions, and the enrichment of core housekeeping genes in genomic regions that were most deficient in PAVs (table 1). Compared with tissue-specific genes, housekeeping genes generally evolve more slowly, have lower Ka/Ks ratio (the rate ratio of nonsynonymous to synonymous substitutions), and tend to evolve under strong purifying selection (Zhang and Li 2004). Among the regions deficient in PAVs, classical housekeeping genes (Zhang and Li 2004) including tRNAs, ribosomal proteins, elongation factors, NAD+ transporters, transcription factors, homeobox proteins were highly enriched (supplementary tables S2 and S3, Supplementary Material online).

In contrast to this, when we used a traditional method to detect evidence of purifying selection on weakly deleterious alleles based on an excess of low-frequency variants in the allele frequency spectrum, we found no such evidence (supplementary fig. S6, Supplementary Material online). This approach is based on the expectation that selection limits the spread of deleterious variants in the population. In our analysis, we found the opposite trend, in which genic PAVs were underrepresented in the lowest frequency bin relative to nongenic variants. This may be because PAVs in genes tend to be strongly deleterious rather than mildly deleterious. Although the efficiency of selection is expected to be reduced on weakly deleterious variants in selfing species (Schemske and Lande 1985; Charlesworth et al. 1990; Charlesworth and Wright 2001) and this has been shown empirically in *A. thaliana* and related plant species (Slotte et al. 2013; Slotte 2014; Laenen et al. 2018), purifying selection on loci that are lethal in the homozygous state is expected to be strong in selfing species because these variants are quickly exposed to selection (Stebbins 1950; Shields 1982; Schemske and Lande 1985; Glémin 2007; Charlesworth and Willis 2009; Arunkumar et al. 2015). Further, the overall pattern that we observe in which genic regions are more highly conserved than nongenic regions is consistent with the expected effects of background selection (Charlesworth et al. 1993) and that linkage disequilibrium is increased within genes in *A. thaliana* (Berger et al. 2015). Taken together our results suggest that newly arising genic PAV mutations tend to be strongly deleterious and therefore often evolve under purifying selection in *A. thaliana*, whereas those that remain in the population do not evolve under strong purifying selection relative to intergenic PAVs.

However, some categories of genes were much more likely to contain PAVs relative to the genomic background. We found that defense-related genes (R-genes, secondary metabolites) and F-box genes have an excess of PAVs compared with other genes. R-genes and F-box genes are multigene families known to be rapidly evolving (Xu et al. 2009; Yang et al. 2013). Further, plants produce a massive number of metabolites and only a few of these are primary (those common to all organisms); others are known as secondary metabolites (Pichersky and Gang 2000; Labarrere et al. 2019). Many secondary metabolites are thought to be involved in defense against herbivores and pathogens (Isah 2019) and previous work has shown that a subset of these regions that are involved in the production of glucosinolates appears to be rapidly evolving (Kliebenstein et al. 2001; Kliebenstein 2004) that multiple losses have occurred over evolutionary time across Eurasia. (Katz et al. 2020). Our results agree with these findings, and provide evidence that these genes are not only rapidly evolving but also belong to the dispensable genome and carry high levels of structural variation.

We further found that PAVs in defense response genes tend to be present at more intermediate frequencies within populations compared with the genomic background (fig. 4), suggesting they are maintained in populations by some form of balancing selection. This maintenance of polymorphism can involve spatially- or temporally varying selection and/or fitness trade-offs. The classical gene-for-gene model posits that a specific gene (R-gene) from the host is involved in recognition of a specific pathogen avirulence (avr) gene (Flor 1971). In many cases, the gene-for-gene model in plants can explain maintenance of polymorphism in the evolution of disease resistance genes (Bergelson et al. 2001; Tian et al. 2002; Gao et al. 2009; Karasov et al. 2014). However, it has been shown that R-gene polymorphism in *A. thaliana* is sometimes more complex (Karasov et al. 2014; Laflamme et al. 2020). The panNLRome in *A. thaliana* recently showed that although there is high variation in NLR genes this diversity is not unlimited (Van de Weyer et al. 2019). Trade-offs between growth and herbivore or pathogen resistance (Coley et al. 1985; Walling 2009; Huot et al. 2014) also likely contribute to the maintenance of polymorphisms in a population. For example, a hyperactive *ACD6* allele is known to strongly increase resistance of *A. thaliana* to a broad range of pathogens but alters its growth dramatically (Todesco et al. 2010). An additional mechanism that acts to maintain variation in R-genes involves interactions between incompatibility alleles. Several gene combinations, especially for disease resistance genes, are reported as lethal for the plants (Bomblies et al. 2010; Smith et al. 2011; Chae et al. 2014; Tran et al. 2017). This phenomenon involves autoimmunity and hybrid necrosis, which is the opposite of heterosis or hybrid vigor (Chae et al. 2014; Tran et al. 2017). Further, accumulating genomic data from related species suggests that balancing selection may be common in defense response loci in other species as well (Koenig et al. 2019).

The effects of SVs on fitness in the wild may change across environments or over time, as has been shown in previous focused studies (Gao et al. 2009; Huard-chauveau et al. 2013). Recently, it was further shown that loss of function variants may contribute to local adaptation and phenotypic diversity in *A. thaliana* (Monroe et al. 2018; Xu et al. 2019). Consistent with this, we found strong associations between several genic PAVs and environmental variables, including several involved in response to vernalization with minimum temperature in the coldest month *ELF9* (AT5G16260), *AGL31*(AT5G65050), and *MAF3* (AT5G65050) (fig. 6). This is reminiscent of the patterns observed in natural populations at the well-known *FRIGIDA* locus, where loss of function alleles obviate the need for cold exposure before flowering (Le Corre et al. 2002; Stinchcombe et al. 2004; Shindo et al. 2005; Zhang and Jiménez‐Gómez 2020).

The set of SVs identified and genotyped here will be useful alone or in combination with available SNP data to investigate *A. thaliana* evolution and trait architecture using GWAS or recombinant populations.

## Materials and Methods

### Samples

We retrieved Illumina short-read data for 1,327 samples from four studies (Alonso-Blanco et al. 2016; Durvasula et al. 2017; Zou et al. 2017; Fulgione et al. 2018). We excluded 26 samples from the analysis due to low data quality, so that the final data set included 1,301 samples. Besides publicly available data, we also sequenced one sample (PRJNA638240) from Cape Verde Island (Cvi-0) with Pacific Biosciences long-read sequencing technology (PacBio) (supplementary table S1, Supplementary Material online). For this, we sterilized and sowed Cvi-0 seeds on MS (Murashige & Skoog) media supplemented with sucrose. Then we stratified seeds for 6 days. Later we moved seeds to a growth chamber for 2 weeks. Finally, we transferred plants to the dark, where they remained for 3 days before DNA extraction using a NucleoSpin plant II protocol. After quality checks, size selection was performed with a Blue Pippin (Sage Science) (>10 kb) and DNA sequencing was performed with PacBio RS II. DNA extraction, size selection, and PacBio sequencing was performed at Max Planck Genome Center in Cologne, Germany.

### Performance Comparison of SV Callers

We tested three popular tools designed for SV identification from short-read data to compare their performance. These included LUMPYEXPRESS (v0.2.13) (Layer et al. 2014), DELLY (v0.8.1) (Rausch et al. 2012), and PINDEL (v0.2.5b8) (Ye et al. 2009). LUMPYEXPRESS is an automated breakpoint detection tool for standard analysis which internally uses LUMPY (v0.2.13) (Layer et al. 2014). Although LUMPYEXPRESS (v0.2.13) uses three different sources of information including Read Pair (RP), Split Read (SR), and Read Depth (RD) information, DELLY (v0.8.1) uses only RP and SR information and PINDEL (v0.2.5b8) relies on only SR information to identify structural variations.

To identify the best software for calling SVs in short-read data, we used two approaches: a simulation-based approach and a comparison to calls from long-read data. For the simulation approach, we introduced 1,000 structural variations (maximum length 10 kb and SNP mutations frequency 0.1) with SURVIVOR (v1.0.6) (Jeffares et al. 2017) on the *A. thaliana* TAIR10 genome for each kind including deletions, tandem duplications, and inversions. We simulated NGS reads by WGSIM (v1.9) (Li et al. 2009) using the following parameters (-h –N 10000000 -1 150 -2 150) from SVs introduced reference. Later, we mapped the simulated reads to original TAIR10 by BWA-MEM (v0.7.17) (Li and Durbin 2009) with default parameters. Finally, we called SVs using LUMPYEXPRESS (v0.2.13), DELLY (v0.8.1), and PINDEL (v0.2.5b8) with default parameters.

For the PacBio approach, we first mapped PacBio reads to TAIR10 using NGMLR (v0.2.7) (Sedlazeck et al. 2018) with default settings. After mapping, we converted SAM (the Sequence Alignment/Map format) files to BAM (Binary Alignment/Map format) files by SAMTOOLS (v1.8) (Li et al. 2009) with the parameter settings “view -Sb.” Then, we employed SNIFFLES (v1.0.8) (Sedlazeck et al. 2018) the settings “–genotype, -l 50” to call SVs. Finally, we compared the performance of the three short-read tools based on SNIFFLES (v1.0.8) calls. For both approaches, we considered a minimum of 50% reciprocal overlap to represent true positives then we calculated their sensitivity (True Positives/[True Positives + False Negatives]) and precision (True Positives/[True Positives + False Positives]).

### Calling of SVs and SNPs in Natural Populations

We identified SVs among 1,301 accessions using the pipeline (supplementary fig. S1, Supplementary Material online, https://github.com/HancockLab/SVS_A.thaliana) that consists of LUMPYEXPRESS (v0.2.13), SVTYPER (v0.7.0) (Chiang et al. 2015), and SVTOOLS (v0.4.0) (https://doi.org/10.5281/zenodo.1442926, last accessed February 10, 2019). Our pipeline is forked and modified from (https://github.com/arq5x/lumpy-sv, last accessed December 3, 2018). After calling SVs (deletions, tandem duplications, and inversions), we performed two genotyping steps. First, we conducted individual genotyping with SVTYPER (v0.7.0), and then we conducted joint genotyping with SVTOOLS (v0.4.0). This final genotyping allowed us to differentiate missing genotypes from matches to the reference. The discovery set can have an influence on the power to identify variants across populations. Therefore, we included all individuals in the discovery panel to reduce the false-negative rate overall; however, this likely results in a bias towards higher SV discovery rates in deeply sampled populations and regions.

Our pipeline failed for 26 samples, which were thus excluded from the analysis. There was no clear reason why these samples failed, and as they are not clustered into any one population removing them is not expected to bias the data set. These samples include (collection location, sequencing facility) are BRR4 (MPI Tübingen), BRR12 (MPI Tübingen), BRR57 (MPI Tübingen), BRR107 (MPI Tübingen), Bur-0 (IRL, Mott), Can-0 (ESP, Mott), Dem-4 (Salk), KBS-Mac-74 (MPI Tübingen), LI-SET-036 (MPI Tübingen), MSGA-61 (MPI Tübingen), Oy-0 (Mott), Sf-2 (ESP, Mott), Paw-13 (MPI Tübingen), Paw-20 (MPI Tübingen), Rsch-4 (RUS, Mott), Yng-53 (MPI Tübingen), Tsu-0 (Mott), Uod-7 (AUT, Salk), Yng-4 (MPI Tübingen), Zu-0 (SUI, Mott), 11PNA1.14 (MPI Tübingen), 328PNA062 (MPI Tübingen), 87 (CHN, Yangtze Genomes), 36-31 (CHN, Yangtze Genomes), 36-17 (CHN, Yangtze Genomes), and 27-9(CHN, Yangtze Genomes).

The VCF file used for subsequent population genetic analysis was generated by setting an upper size limit of 10 kb, including only polymorphic PAVs. Therefore, the sizes of variants included in this analysis range from 1 bp to 10 kb. The nature of short-read data prevented us from identifying large SVs. We provide two VCF files: one with filtering applied with only PAV variants included, and one with all raw calls including PAVs, inversions, and tandem duplications.

In addition to PAVs, we also called biallelic SNPs for all 1,301 samples following the same pipeline we used previously (Durvasula et al. 2017; Fulgione et al. 2018).

### Examining Population Structure Using PAVs

We produced a whole-genome neighbor-joining (NJ) tree in R with the ape (v5.0) package (Paradis and Schliep 2019) using the same set of samples used in (Durvasula et al. 2017) except for herbarium samples, which were sequenced with single-end sequencing data and could therefore not be included in our SV-calling pipeline.

### Identifying Genomic Hotspots and Conserved Regions

We defined genic and nongenic regions based on TAIR10 annotation. Any region that overlaps with a gene is treated as a genic region and the rest is treated as a nongenic region. We compared genic and nongenic regions to see if there is any difference based on PAVs. We found more PAVs in intergenic regions compared with genic regions. To test the significance of finding a higher proportion of PAVs in intergenic regions than genic regions, we used the χ^2^ test. We set the expected genic region to 60,104,494 bases (sum of all genes length without overlap) and expected intergenic region to 59,041,854 bases (sum of all nongenic regions). If the distribution of PAVs throughout the genome was random, we would not expect to see any difference between genic and intergenic regions. Our observations for genic regions and intergenic regions overlapping with PAVs were 8,920,166 bases and 30,766,220 bases, respectively.

Later we focused on the genic regions to be able to see the gene sets with excesses or deficits of PAVs. Genes that overlap with PAVs were extracted and performed enrichment analysis. Same enrichment analysis was also done for the genes that never overlap with a PAV. We tested for GO term (Carbon et al. 2019) enrichment analysis using one-tailed FET with Benjamini correction as well as Gowinda (v1.0), a more conservative test that takes gene size and clustering into account using a permutation-based approach to assess significance (Kofler et al. 2012).

We calculated unfolded SFS for each population and genomic class (whole genome, intergenic, genic, and defense genes). To produce the frequency spectrum for each population, we calculated proportion of variants that were present once in the population (i.e., singletons), twice (i.e., doubletons), etc. and these are plotted in figure 4 and supplementary figures S7 and S8, Supplementary Material online. The frequency spectra were polarized to a consensus ancestral genome. This consensus ancestral genome was created from five accessions chosen to represent distinct diverged *A. thaliana* lineages including (Lebanon [Qar-8a], Italy [Etna-0], Madeira [12761], North Middle Atlas [22000], and High Atlas [18511]) based on analyses in previous study (Durvasula et al. 2017). This set represents the major “relict” lineages of *A. thaliana*. We limited the set to five samples because only a single “relict” sample was available from the Levant (Lebanon) region. For each PAV, we calculated allele frequency among these five accessions. The highest frequency allele for each locus was used as the ancestral state. NA was assigned to the ancestral state where data were missing for more than one individual or when the allele frequency is equal to 0.5.

The samples that we retrieved from the 1001 Genomes project were separated into populations based on their admixture groups (Alonso-Blanco et al. 2016). Other samples were grouped based on their geographical origins including Madeira, China (Yangtze River Basin and North-Western China), and Morocco (High Atlas, South Middle Atlas, North Middle Atlas, and Riff).

We found a clear shift to the intermediate frequencies for PAVs on defense response genes. To test whether there was a statistically significant excess of intermediate frequency PAVs in defense response genes relative to the genome-wide distribution, we calculated the Tajima’s *D* statistic for the set of defense genes in each population and compared this with the Tajima’s *D* statistic for the entire genome. We found that for each population, Tajima’s *D* was more positive for defense genes compared with the total set of genes. To assess significance for this result, we randomly subsampled genic PAVs to match the number of defense response PAVs 100,000 times and calculated an empirical *P* value based on the distribution of Tajima’s *D* in the resampled data sets. We further examined evidence for balancing selection using BetaScan, which relies on a signal of high variation at the haplotype level (Siewert and Voight 2017, 2020). After employment of BetaScan, we extracted 5% extreme tail of highest β scored SNPs and performed enrichment analysis with FET. We found significant enrichment of defense response genes for all groups.

### Correlation with Environmental Variables (GWAS)

We used the linear mixed model association method, GEMMA (v0.98.1) (Zhou and Stephens 2012), to assess correlation between PAVs and SNPs with environmental variables. Environmental variables were obtained as geoTiff files from WorldClim2 (Fick and Hijmans 2017). We extracted data for four environmental variables (geoTiff files) using the raster package in R. Then we used geo-referencing information to compute the values for each accession with the “extract” function from the raster package. These computed values were treated as phenotypes for association mapping. Individual samples that are genetically and environmentally divergent from the bulk of samples violate assumptions of the linear mixed model approach. Thus, the following divergent samples were removed before conducting GWAS (6911, 9762, 9764, 10024, 35520, 12761, 12672, 12763, 12908, 22017, 22019, 22022, 22638, and 27153) to avoid effects of outliers in the LMM analysis. To prepare files for GEMMA (v0.98.1), we converted the VCF file to plink file with VCFTOOLS (v0.16) (Danecek et al. 2011) (–plink) and then ran PLINK (v2.0) (Purcell et al. 2007) to create a bed file (–make-bed). Next, we used GEMMA (v0.98.1) to calculate the kinship matrix (-gk 1, -maf 0.1) and ran GEMMA (v0.98.1) under the linear mixed model with a minor allele frequency cut-off of 10% (-lmm 2, -maf 0.1). The minor allele frequency cutoff acts to remove outliers that could otherwise drive signals in the analysis. To prioritize candidate functional PAVs, we focused on genes within 10 kb of a given climate-associated PAV. In order to examine how well SNPs could represent climate-correlated PAVs, we estimated linkage disequilibrium (LD) between all SNPs within 10 kb of a PAVs with a GWAS *P* value <0.001 using PLINK (v2.0) with the command –r2 –ld-window-kb 10 –ld-window 999999 and filtered for the PAVs of interest for each environmental GWAS. For each PAV, we identified the SNP with the highest *r*^2^, created histograms for these, and used the distribution to estimate the mean and SD of *r*^2^ between these PAVs and the most correlated SNPs.

## Supplementary Material

Supplementary data are available at *Molecular Biology and Evolution* online.

## Supplementary Material

msaa309_Supplementary_Data
