# Supplementary material for: A New Catalog of Structural Variants in 1,301 A. thaliana Lines from Africa, Eurasia, and North America Reveals a Signature of Balancing Selection at Defense Response Genes
Source: Mol Biol Evol. 2020 Nov 28;38(4):1498–511. doi: 10.1093/molbev/msaa309 (PMC8042739; doi:10.1093/molbev/msaa309)
Supplement: msaa309_Supplementary_Data [file msaa309_supplementary_data.pdf]

Supplementary Information

A new catalogue of structural variants in 1301 *A. thaliana* lines from Africa, Eurasia and North America reveals a signature of balancing at defense response genes

Mehmet Göktay[1], Andrea Fulgione[1], Angela. M. Hancock[1,2]

[1] Max Planck Institute for Plant Breeding Research, Cologne, Germany

[2] Corresponding author: Angela M. Hancock, hancock@mpipz.mpg.de

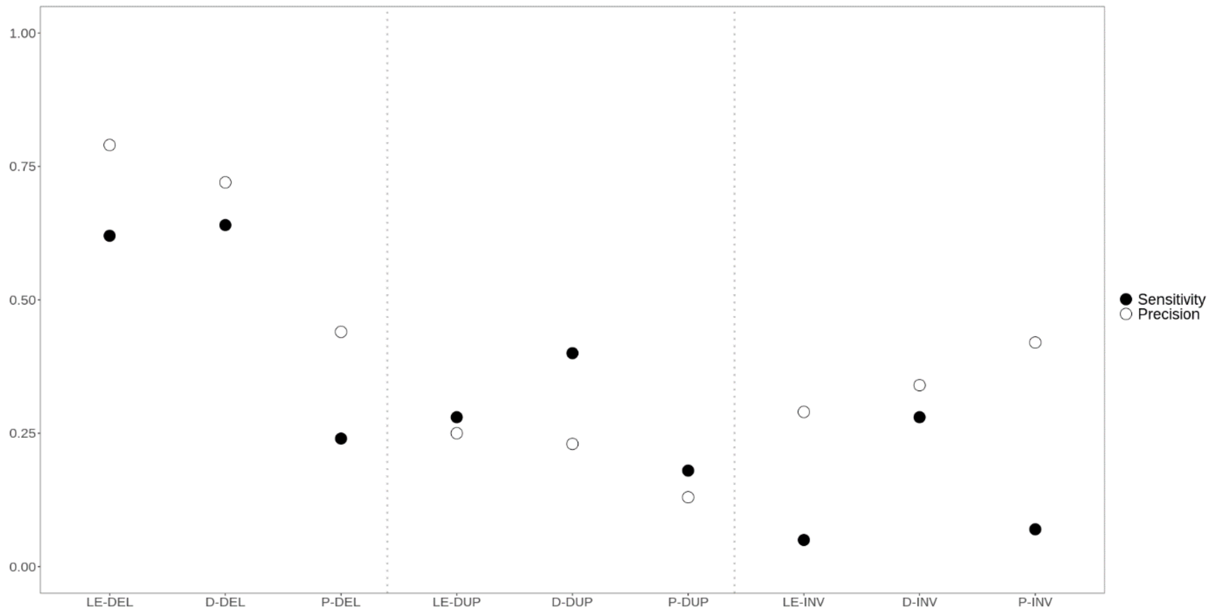

Supplementary Figure S1. Sensitivity and precision of short read SV calling results with three different callers under the assumption that SV calls from PacBio data represent true SVs. Abbreviations along the x-axis represent the combination of method used and variant type and are defined as follows: LumpyExpress (LE), Delly (D), Pindel (P), Deletion (DEL), Duplication (DUP), Inversion (INV)).

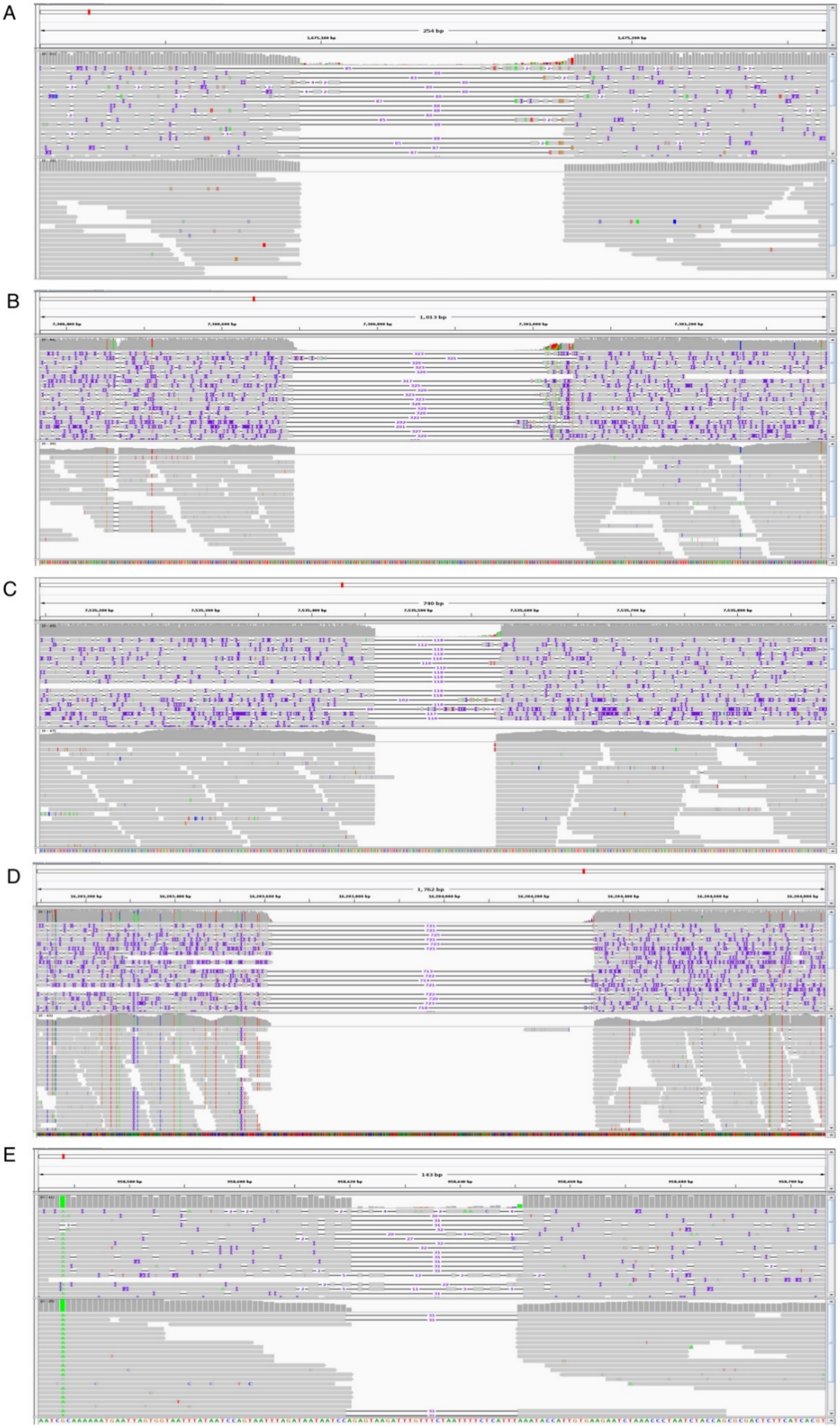

Supplementary Figure S2. Randomly selected examples of PAVs found with both PacBio (long read) and Illumina (short read) data visualized with Integrative Genomics Viewer (IGV). For each panel, the top alignment shows PacBio reads and bottom alignment shows Illumina reads for the Cvi-0 accession. Colored vertical lines indicate SNPs and Indels. SNPs are colored as green, red, blue and orange for Adenine, Thymine, Cytosine and Guanine respectively. Purple color indicates insertions and purple numbers indicate the insertion length. A) Chr5:1675093-1675181 B) Chr5:7300694-7301054 C) Chr2:7535460-7535573 D) Chr3:16203615-16204336 E) Chr1:958620-958652.

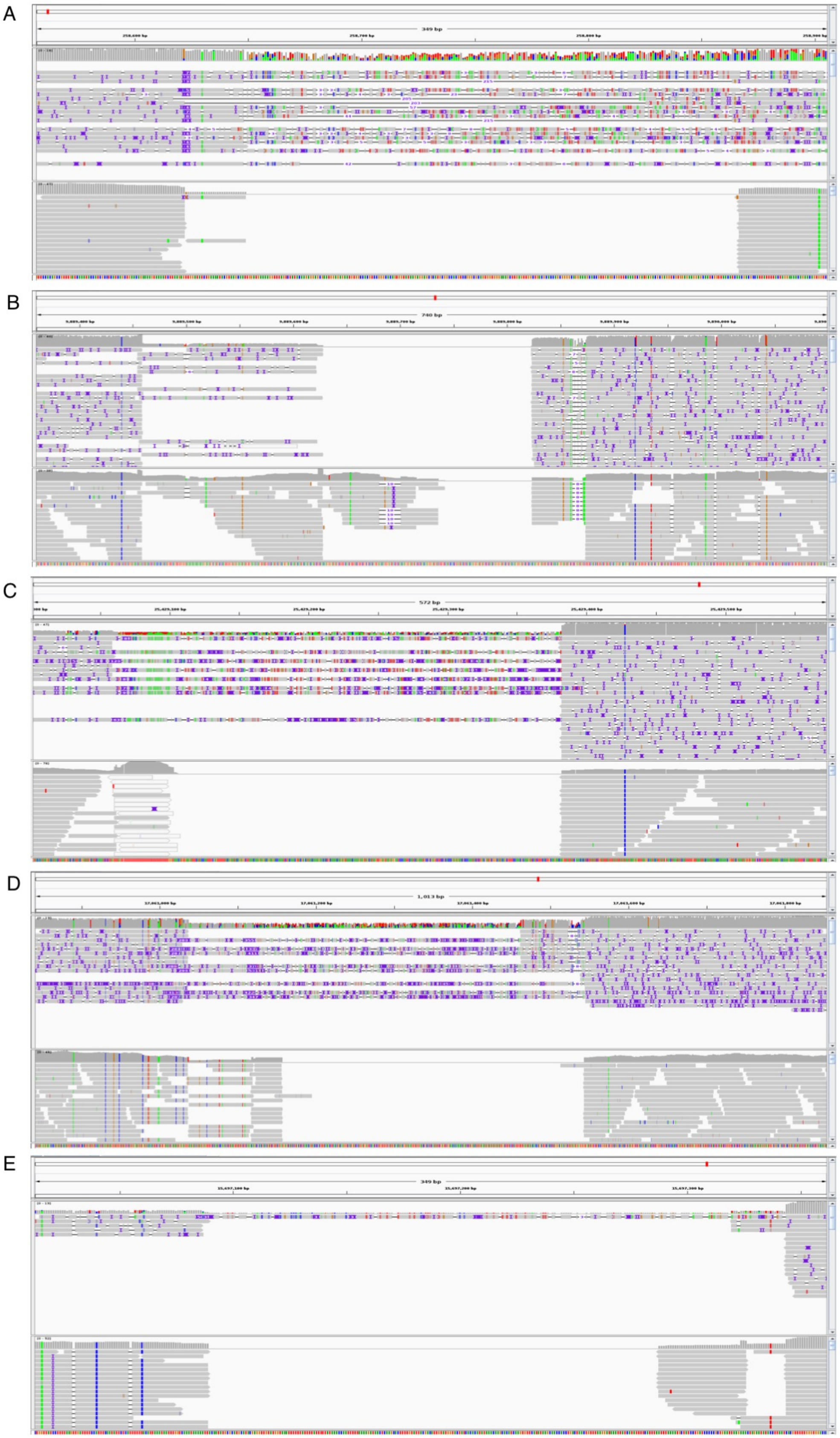

Supplementary Figure S3. Randomly selected examples of PAVs found with Illumina (short read) data but not with PacBio (long read) visualized with Integrative Genomics Viewer (IGV). For each panel, the top alignment shows PacBio reads and bottom alignment shows Illumina reads for the Cvi-0 accession. Colored vertical lines indicate SNPs and Indels. SNPs are colored as green, red, blue and orange for Adenine, Thymine, Cytosine and Guanine respectively. Purple color indicates insertions and purple numbers indicate the insertion length. A) Chr4:258648-258865 B) Chr2:9889714-9889872 C) Chr1:25429062-25429382 D) Chr5:17061156-17061543 E) Chr4:15697089-15697287.

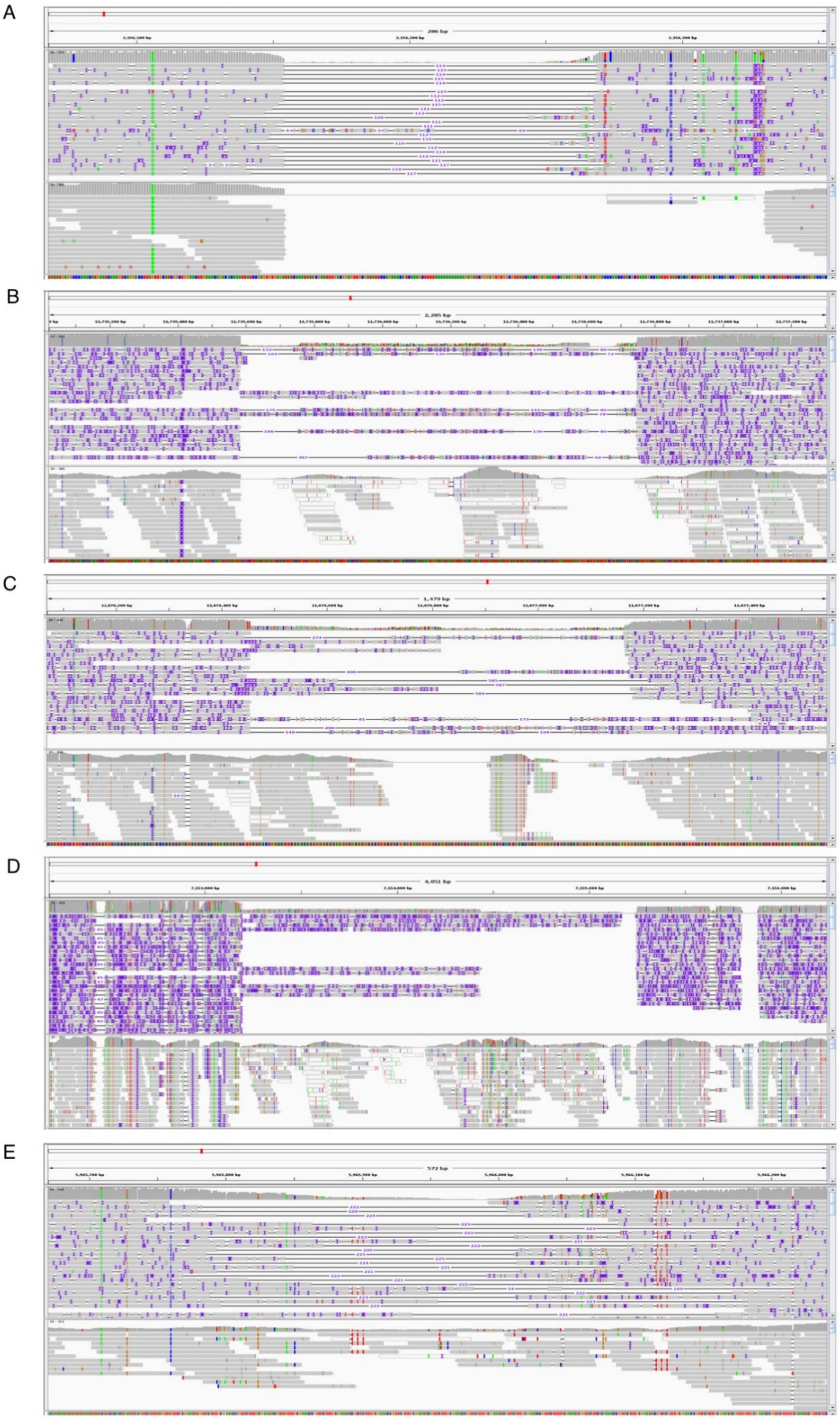

Supplementary Figure S4. Randomly selected examples of PAVs found with PacBio (long read) but not with Illumina (short read) data visualized with Integrative Genomics Viewer (IGV). For each panel, the top alignment shows PacBio reads and bottom alignment shows Illumina reads for the Cvi-0 accession. Colored vertical lines indicate SNPs and Indels. SNPs are colored as green, red, blue and orange for Adenine, Thymine, Cytosine and Guanine respectively. Purple color indicates insertions. A) Chr1:2156154-2156268 B) Chr1:11735586-11736749 C) Chr2:11076457-11077165 D) Chr5:7153193-7155249 E) Chr1:5965844-5966068.

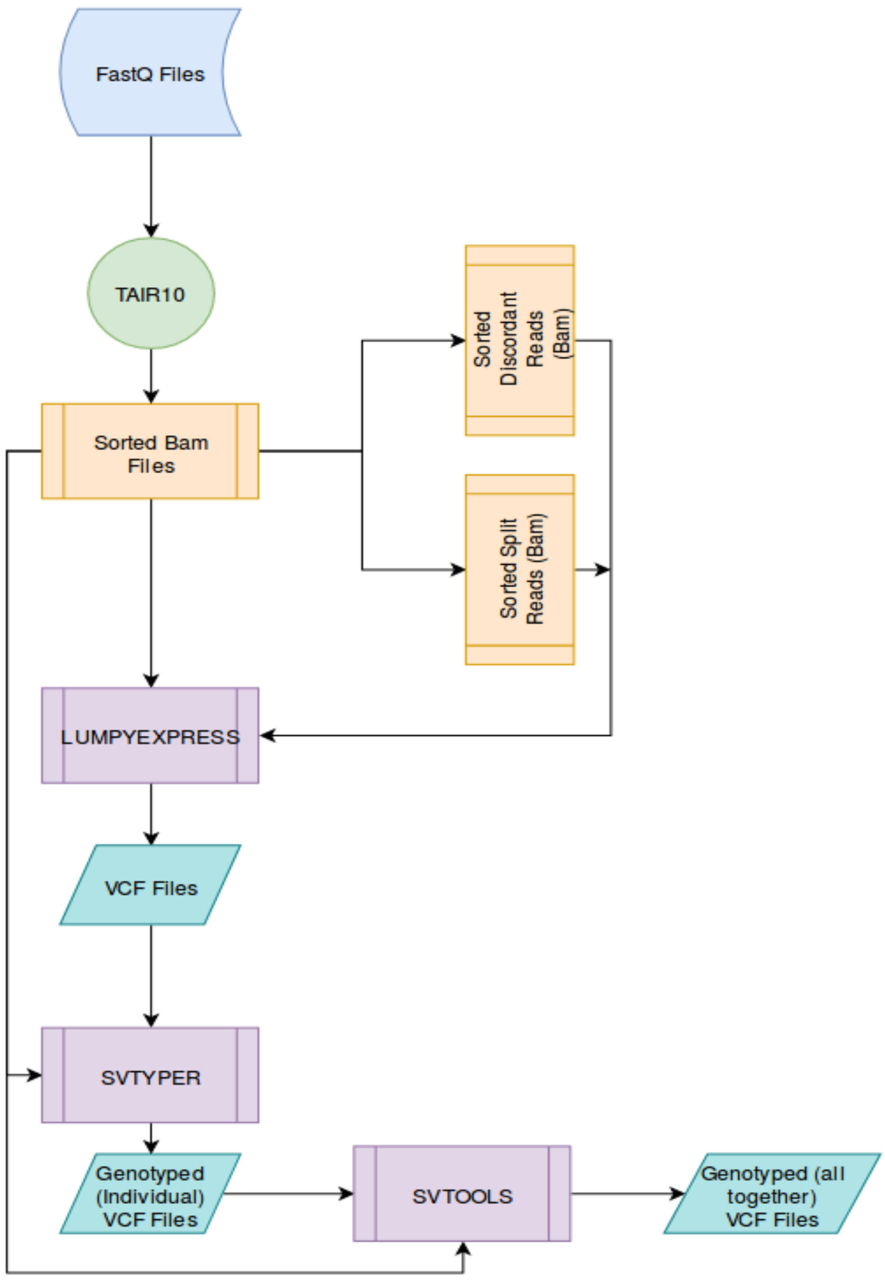

Supplementary Figure S5. Pipeline used for SV identification and genotyping across the total set of 1301 diverse *A. thaliana* accessions

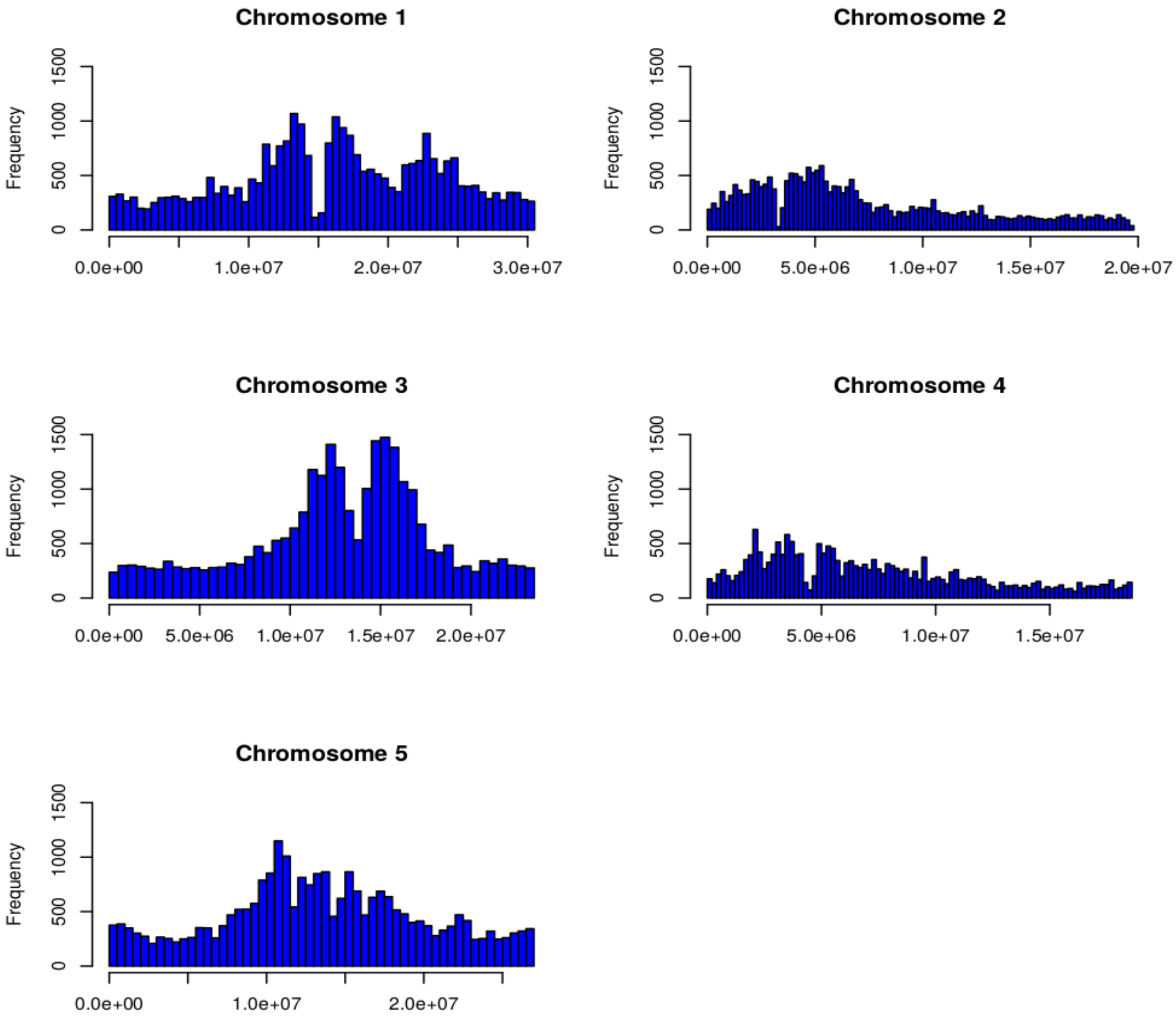

Supplementary Figure S6. Distribution of PAVs identified in 1301 accessions across the five *A. thaliana* chromosomes.

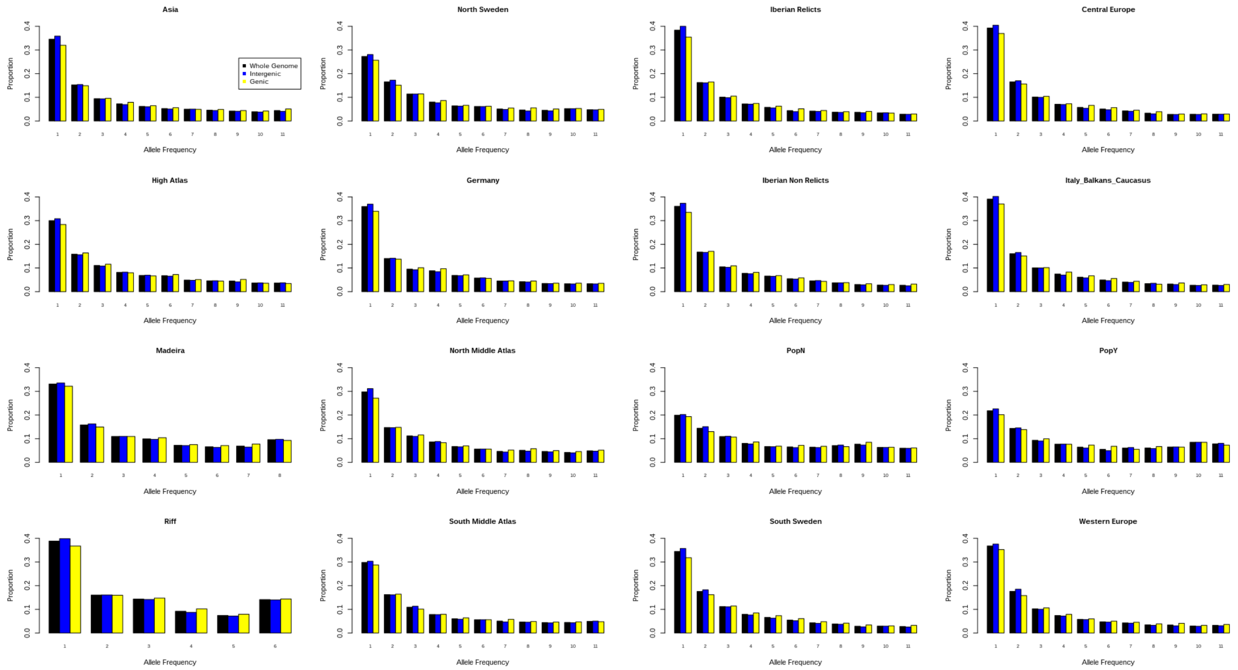

Supplementary Figure S7. Site frequency spectra (SFS) for PAVs including whole genome, genic, intergenic and for all populations. Colors represent site frequency spectra across the entire genome (black), intergenic PAVs (blue), and genic PAVs (yellow).

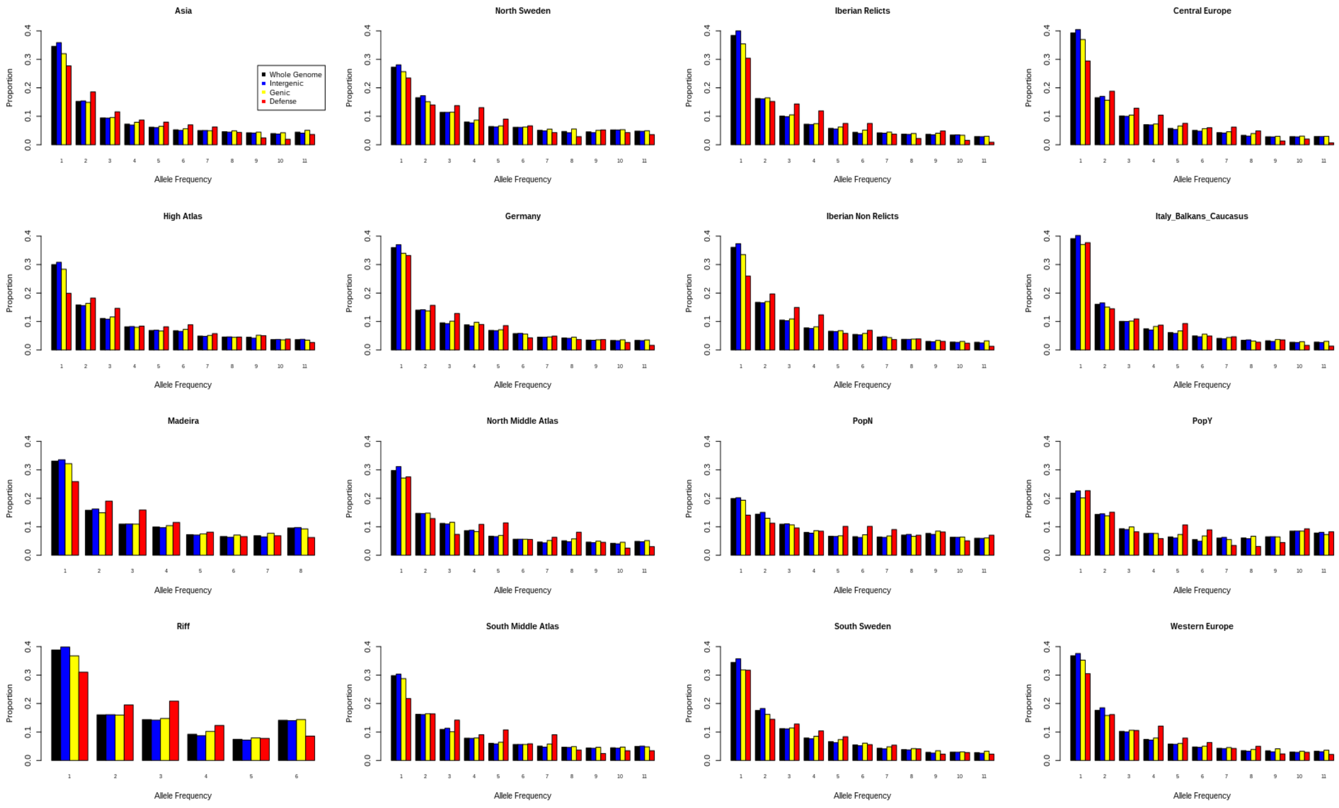

Supplementary Figure S8. Site frequency spectra (SFS) for PAVs including whole genome, genic, intergenic and defense genes for all populations. Colors represent site frequency spectra across the entire genome (black), intergenic PAVs (blue), genic PAVs (yellow) and genes involved in defense (red).

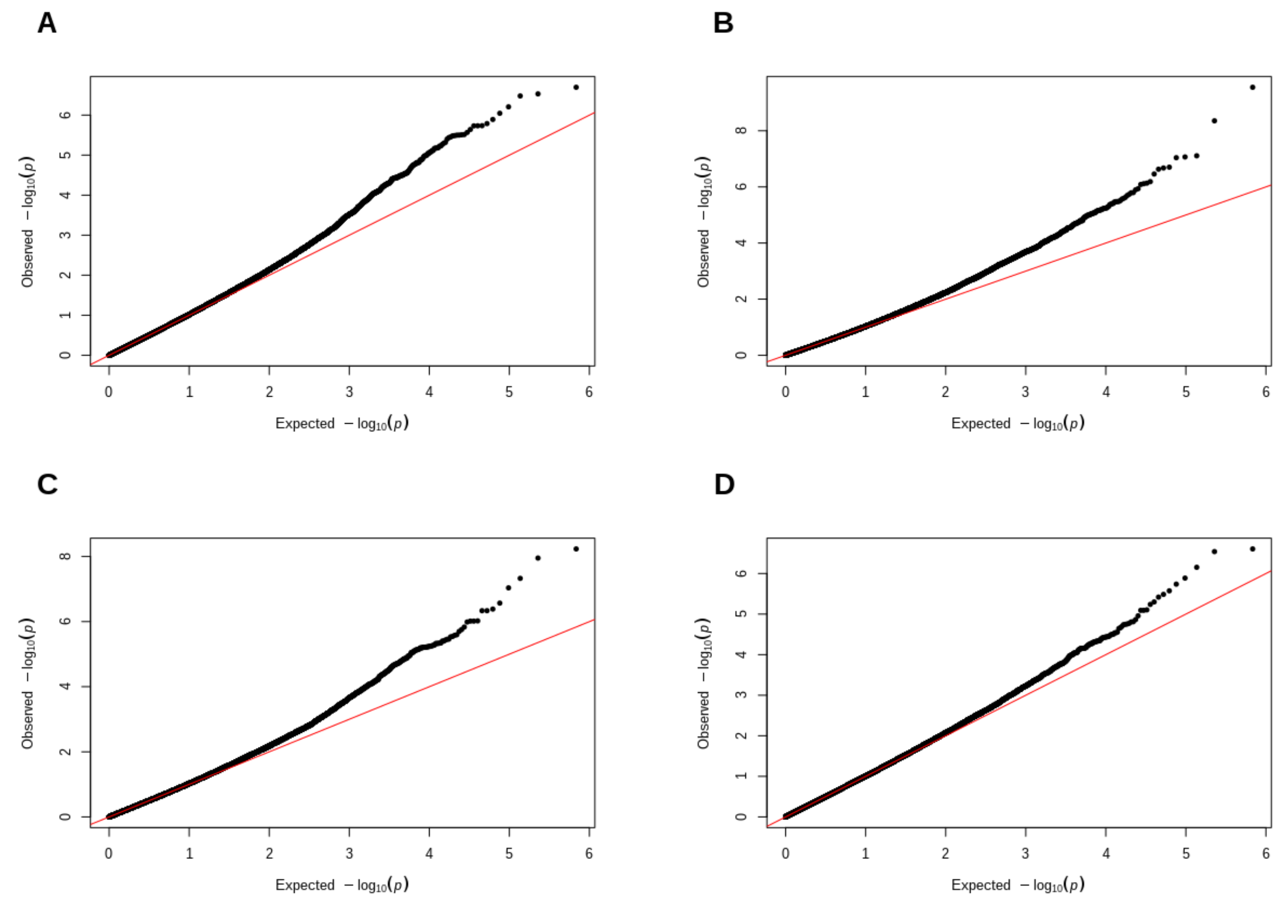

Supplementary Figure S9. QQ Plots for SNPs. (A) Bio5: Maximum temperature of the warmest month, (B) Bio 6: Minimum temperature of the coldest month, (C) Bio13: Precipitation of the wettest month, and (D) Bio14: Precipitation of the driest month.

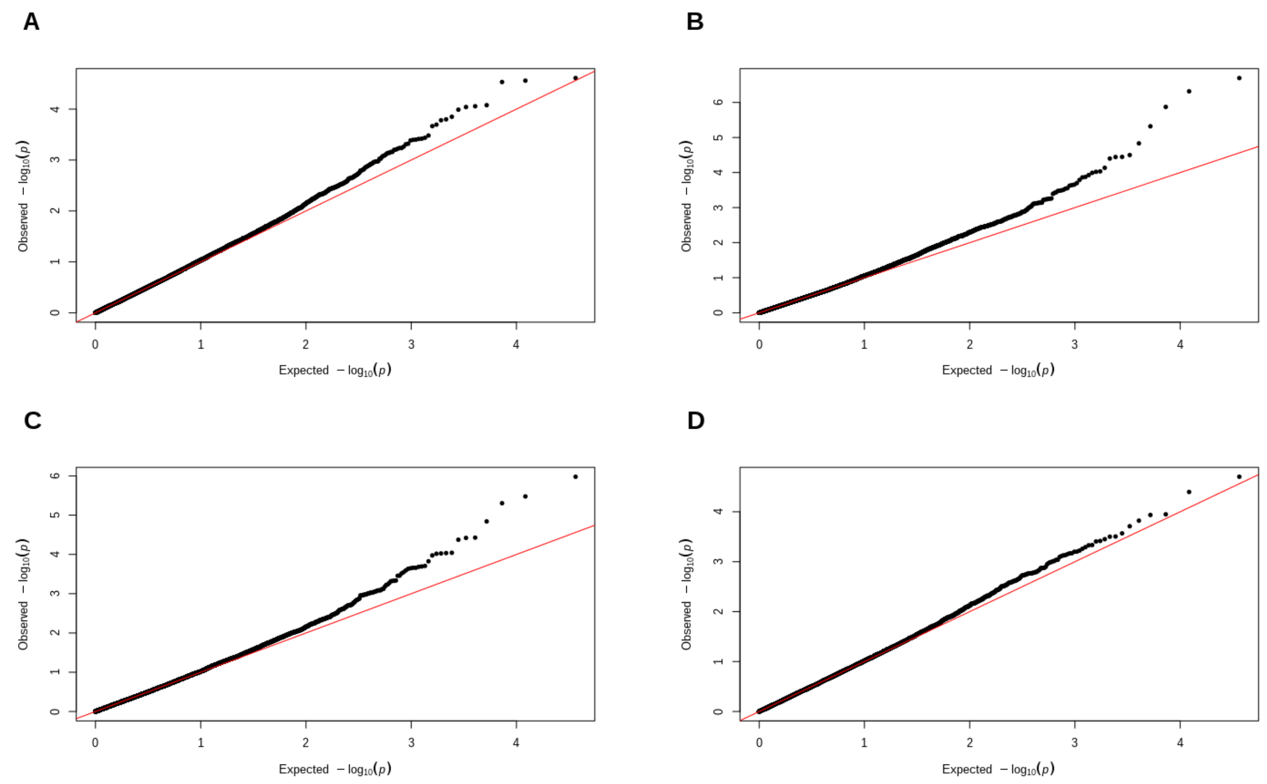

Supplementary Figure S10. QQ Plots showing the relationship between observed and expected environmental association p-values with PAVs. (A) Bio5: Maximum temperature of the warmest month, (B) Bio 6: Minimum temperature of the coldest month, (C) Bio13: Precipitation of the wettest month, and (D) Bio14: Precipitation of the driest month

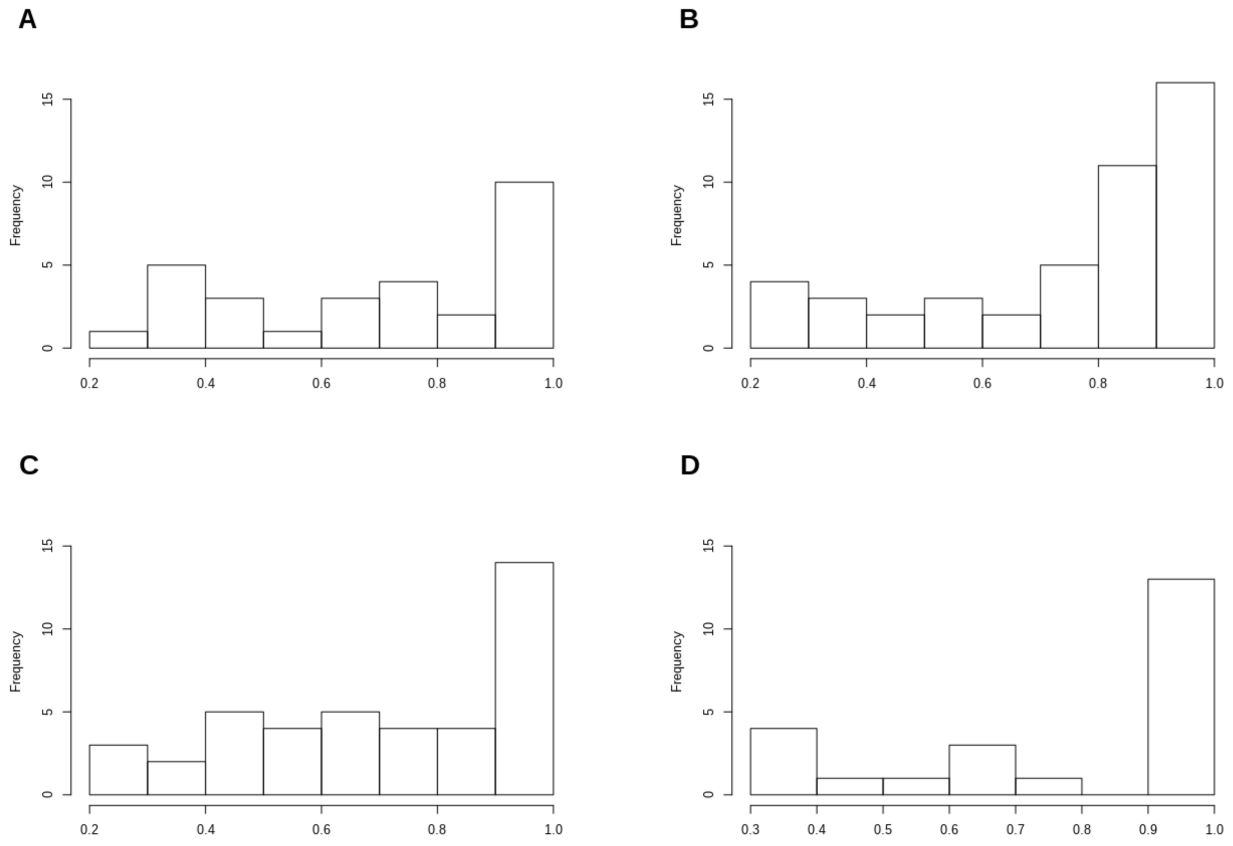

Supplementary Figure S11. Linkage disequilibrium between PAVs with p-values less than 0.001 and SNPs. For each PAV, the SNP with the highest LD was identified and the distributions of r^2 are plotted. Bio5: Maximum temperature of the warmest month, (B) Bio6: Minimum temperature of the coldest month, (C) Bio13: Precipitation of the wettest month, and (D) Bio14: Precipitation of the driest month.

Supplementary Table S1: Summary statistics of Cvi-0 PacBio sequencing data

Mean read length	6220.8
Mean read quality	8.2
Median read length	4701
Median read quality	8.4
Number of reads	1045431
Read length (N50)	10279
Total bases	6503464315
Genome coverage (135 Mb)	54.1x

Supplementary Table S2: One-tailed Fisher's exact test for all PAVs.

GO_term_id	P-value	Benjamini-corrected P-value	GO term
GO:0006414	4.10E-45	1.10E-41	Translational elongation
GO:0006412	3.90E-39	5.40E-33	Translation
GO:0000154	1.70E-10	1.60E-07	rRNA modification
GO:0035195	2.40E-07	1.70E-04	Gene silencing by miRNA
GO:0010200	6.00E-06	3.40E-03	Response to chitin
GO:0006355	1.40E-05	6.50E-03	Regulation of transcription, DNA-templated
GO:0045168	6.10E-05	2.40E-02	Cell-cell signaling involved in cell fate commitment

Supplementary Table S3: Permutation-based test for all PAVs

GO_term_id	P-value	Benjamini-corrected P-value	GO term
GO:0050794	1.00E-05	9.13E-05	Regulation of cellular process
GO:2000112	1.00E-05	9.13E-05	Regulation of cellular macromolecule biosynthetic process
GO:0050789	1.00E-05	9.13E-05	Regulation of biological process
GO:0009725	1.00E-05	9.13E-05	Response to hormone stimulus
GO:0009719	1.00E-05	9.13E-05	Response to endogenous stimulus
GO:0051252	1.00E-05	9.13E-05	Regulation of RNA metabolic process
GO:0009743	1.00E-05	9.13E-05	Response to carbohydrate stimulus
GO:0009739	1.00E-05	9.13E-05	Response to gibberellin stimulus
GO:0051171	1.00E-05	9.13E-05	Regulation of nitrogen compound metabolic process
GO:0042221	1.00E-05	9.13E-05	Response to chemical stimulus
GO:0034645	1.00E-05	9.13E-05	Cellular macromolecule biosynthetic process
GO:0060255	1.00E-05	9.13E-05	Regulation of macromolecule metabolic process
GO:0009889	1.00E-05	9.13E-05	Regulation of biosynthetic process
GO:0071702	1.00E-05	9.13E-05	Organic substance transport
GO:0045449	1.00E-05	9.13E-05	Regulation of transcription
GO:0006412	1.00E-05	9.13E-05	Translation
GO:0031326	1.00E-05	9.13E-05	Regulation of cellular biosynthetic process
GO:0031323	1.00E-05	9.13E-05	Regulation of cellular metabolic process
GO:0010468	1.00E-05	9.13E-05	Regulation of gene expression
GO:0032774	1.00E-05	9.13E-05	RNA biosynthetic process
GO:0010556	1.00E-05	9.13E-05	Regulation of macromolecule biosynthetic process
GO:0010200	1.00E-05	9.13E-05	Response to chitin
GO:0015979	1.00E-05	9.13E-05	Photosynthesis
GO:0019684	1.00E-05	9.13E-05	Photosynthesis light reaction
GO:0006350	1.00E-05	9.13E-05	Transcription
GO:0006351	1.00E-05	9.13E-05	Transcription DNA-dependent
GO:0006355	1.00E-05	9.13E-05	Regulation of transcription DNA-dependent
GO:0006869	1.00E-05	9.13E-05	Lipid transport

GO:0007267	1.00E-05	9.13E-05	Cell-cell signaling
GO:0019219	1.00E-05	9.13E-05	Regulation of nucleobase nucleoside nucleotide and nucleic acid metabolic process
GO:0019222	1.00E-05	9.13E-05	Regulation of metabolic process
GO:0010876	1.00E-05	9.13E-05	Lipid localization
GO:0010033	1.00E-05	9.13E-05	Response to organic substance
GO:0044249	1.00E-05	9.13E-05	Cellular biosynthetic process
GO:0009059	1.00E-05	9.13E-05	Macromolecule biosynthetic process
GO:0009058	1.00E-05	9.13E-05	Biosynthetic process
GO:0080090	1.00E-05	9.13E-05	Regulation of primary metabolic process
GO:0032973	3.00E-05	2.51E-04	Amino acid export
GO:0065007	3.00E-05	2.51E-04	Biological regulation
GO:0032890	3.00E-05	2.51E-04	Regulation of organic acid transport
GO:0051955	3.00E-05	2.51E-04	Regulation of amino acid transport
GO:0051952	3.00E-05	2.51E-04	Regulation of amine transport
GO:0080143	3.00E-05	2.51E-04	Regulation of amino acid export
GO:0019253	3.00E-05	2.51E-04	Reductive pentose-phosphate cycle
GO:0009266	6.00E-05	4.96E-04	Response to temperature stimulus
GO:0009733	8.00E-05	6.48E-04	Response to auxin stimulus
GO:0071843	1.10E-04	9.09E-04	Cellular component biogenesis at cellular level
GO:0070882	1.30E-04	9.69E-04	Cellular cell wall organization or biogenesis
GO:0023060	1.60E-04	1.29E-03	Signal transmission
GO:0023046	1.60E-04	1.29E-03	Signaling process
GO:0071369	2.10E-04	1.67E-03	Cellular response to ethylene stimulus
GO:0009827	2.30E-04	1.85E-03	Plant-type cell wall modification
GO:0022613	2.80E-04	2.19E-03	Ribonucleoprotein complex biogenesis
GO:0009451	3.20E-04	2.59E-03	RNA modification
GO:0042254	3.30E-04	2.63E-03	Ribosome biogenesis
GO:0000160	3.40E-04	2.72E-03	Two-component signal transduction system (phosphorelay)
GO:0009873	3.50E-04	2.78E-03	Ethylene mediated signaling pathway
GO:0007047	3.80E-04	3.00E-03	Cellular cell wall organization

GO:0042787	4.70E-04	3.63E-03	Protein ubiquitination involved in ubiquitin-dependent protein catabolic process
GO:0019685	7.00E-04	5.37E-03	Photosynthesis dark reaction
GO:0009628	7.60E-04	5.80E-03	Response to abiotic stimulus
GO:0009408	8.20E-04	6.11E-03	Response to heat
GO:0023052	9.30E-04	7.03E-03	Signaling
GO:0071669	9.60E-04	7.18E-03	Plant-type cell wall organization or biogenesis
GO:0008150	1.27E-03	9.64E-03	Biological process
GO:0042127	1.33E-03	1.01E-02	Regulation of cell proliferation
GO:0010646	1.43E-03	1.09E-02	Regulation of cell communication
GO:0080167	1.45E-03	1.10E-02	Response to karrikin
GO:0071704	1.49E-03	1.12E-02	Organic substance metabolic process
GO:0015977	1.49E-03	1.12E-02	Carbon fixation
GO:0033036	2.33E-03	1.77E-02	Macromolecule localization
GO:0044260	2.37E-03	1.78E-02	Cellular macromolecule metabolic process
GO:0006091	2.39E-03	1.80E-02	Generation of precursor metabolites and energy
GO:0016117	2.48E-03	1.85E-02	Carotenoid biosynthetic process
GO:0016109	2.48E-03	1.85E-02	Tetraterpenoid biosynthetic process
GO:0051049	2.78E-03	2.10E-02	Regulation of transport
GO:0045912	2.84E-03	2.13E-02	Negative regulation of carbohydrate metabolic process
GO:0043255	2.89E-03	2.16E-02	Regulation of carbohydrate biosynthetic process
GO:0009737	3.03E-03	2.25E-02	Response to abscisic acid stimulus
GO:0009409	3.26E-03	2.38E-02	Response to cold
GO:0006414	3.28E-03	2.39E-02	Translational elongation
GO:0055046	3.53E-03	2.53E-02	Microgametogenesis
GO:0080152	3.55E-03	2.53E-02	Regulation of reductive pentose-phosphate cycle
GO:0080153	3.55E-03	2.53E-02	Negative regulation of reductive pentose-phosphate cycle
GO:0010110	3.55E-03	2.53E-02	Regulation of photosynthesis dark reaction
GO:0016556	3.72E-03	2.65E-02	mRNA modification
GO:0034621	3.84E-03	2.72E-02	Cellular macromolecular complex subunit organization
GO:0048235	4.04E-03	2.82E-02	Pollen sperm cell differentiation
GO:0048766	4.33E-03	3.01E-02	Root hair initiation

GO:0010382	4.43E-03	3.08E-02	Cellular cell wall macromolecule metabolic process
GO:0009987	5.43E-03	3.88E-02	Cellular process
GO:0009834	5.46E-03	3.88E-02	Secondary cell wall biogenesis
GO:0009828	5.51E-03	3.91E-02	Plant-type cell wall loosening
GO:0009061	5.89E-03	4.17E-02	Anaerobic respiration
GO:0016119	5.92E-03	4.18E-02	Carotene metabolic process
GO:0007154	6.19E-03	4.32E-02	Cell communication
GO:0044085	6.28E-03	4.37E-02	Cellular component biogenesis
GO:0006109	6.34E-03	4.40E-02	Regulation of carbohydrate metabolic process
GO:0017004	6.62E-03	4.57E-02	Cytochrome complex assembly
GO:0006825	6.65E-03	4.57E-02	Copper ion transport
GO:0042044	7.11E-03	4.92E-02	Fluid transport
GO:0006833	7.11E-03	4.92E-02	Water transport
GO:0008283	7.27E-03	4.98E-02	Cell proliferation

Supplementary Table S4: Go term enrichment of PAVs for Asia

GO term	P-value	Benjamini-corrected P-value
Signal transduction	6.30E-14	1.40E-10
Defense response	1.0E-10	1.20E-07
Secondary metabolite biosynthetic process	8.10E-08	6.10E-05
SCF-dependent proteosomal ubiquitin-dependent protein catabolic process	1.20E-06	7.10E-04
Terpenoid biosynthetic process	2.30E-06	1.10E-03
Defense response to other organisms	7.60E-05	2.80E-02
Glycosyl compound metabolic process	9.80E-05	3.10E-02

Supplementary Table S5: Go term enrichment of PAVs for Central Europe

GO term	P-value	Benjamini-corrected P-value
Signal transduction	4.40E-13	1.10E-09
Defense response	2.10E-08	2.60E-05
SCF-dependent proteosomal ubiquitin-dependent protein catabolic process	4.00E-06	3.40E-03
Secondary metabolite biosynthetic process	1.40E-05	8.70E-03
Proteolysis	3.00E-05	1.50-E02
Terpenoid biosynthetic process	5.80E-05	2.40E-02
Positive regulation proteosomal ubiquitin-dependent protein catabolic process	9.30E-05	3.30E-02

Supplementary Table S6: Go term enrichment of PAVs for Germany

GO term	P-value	Benjamini-corrected P-value
Signal transduction	2.90E-14	7.10E-11
Defense response	1.30E-11	1.60E-08
Secondary metabolite biosynthetic process	2.90E-05	2.30E-02

Supplementary Table S7: Go term enrichment of PAVs for High Atlas

GO term	P-value	Benjamini-corrected P-value
Signal transduction	2.30E-16	4.00E-13
Defense response	1.00E-13	9.40E-11
Secondary metabolite biosynthetic process	1.60E-05	9.60E-03

Supplementary Table S8: Go term enrichment of PAVs for Iberian non-Relicts

GO term	P-value	Benjamini-corrected P-value
Signal transduction	3.20E-19	7.80E-16
Defense response	6.90E-13	8.30E-10
Flavonoid biosynthetic process	7.00E-06	5.60E-03
Flavonoid glucuronidation	2.00E-05	1.20E-02
Proteolysis	6.20E-05	2.90E-02
Secondary metabolite biosynthetic process	6.20E-05	2.50E-02
Regulation of salicylic acid mediated signaling pathway	1.10E-04	3.80E-02
Terpenoid biosynthetic process	1.40E-04	4.10E-02

Supplementary Table S9: Go term enrichment of PAVs for Iberian Relicts

GO term	P-value	Benjamini-corrected P-value
Signal transduction	8.20E-16	1.70E-12
Defense response	3.90E-13	4.20E-10
Positive regulation of proteosomal ubiquitin-dependent protein catabolic process	5.90E-07	4.20E-04
Secondary metabolite biosynthetic process	6.10E-07	3.30E-04
Terpenoid biosynthetic process	6.00E-05	2.50E-02
Oxidation-reduction process	6.20E-05	2.20E-02
Cell surface receptor signaling pathway	9.90E-05	3.00E-02

Supplementary Table S10: Go term enrichment of PAVs for Italy_Balkans_Caucasus

GO term	P-value	Benjamini-corrected P-value
Signal transduction	5.20E-15	1.30E-11
Defense response	2.20E-12	2.70E-09
Secondary metabolite biosynthetic process	5.80E-06	4.70E-03
Positive regulation of proteasomal ubiquitin-dependent protein catabolic process	2.30E-05	1.40E-02

Supplementary Table S11: Go term enrichment of PAVs for Madeira

GO term	P-value	Benjamini-corrected P-value
Signal transduction	9.20E-15	1.50E-11
Defense response	2.60E-12	2.00E-09
Oxidation-reduction process	7.10E-07	3.80E-04
Secondary metabolite biosynthetic process	8.00E-07	3.20E-02
Flavonoid biosynthetic process	1.50E-04	4.7E-02

Supplementary Table S12: Go term enrichment of PAVs for North Middle Atlas

GO term	P-value	Benjamini-corrected P-value
Signal transduction	2.40E-17	4.20E-14
Defense response	1.70E-16	2.00E-13
Secondary metabolite biosynthetic process	5.70E-06	3.40E-03
Aromatic compound biosynthetic process	3.80E-05	1.70E-02

Supplementary Table S13: Go term enrichment of PAVs for North Sweden

GO term	P-value	Benjamini-corrected P-value
Signal transduction	1.60E-17	3.30E-14
Defense response	2.00E-16	2.30E-13
Secondary metabolite biosynthetic process	4.90E-07	3.30E-04

Supplementary Table S14: Go term enrichment of PAVs for PopN

GO term	P-value	Benjamini-corrected P-value
Signal transduction	2.20E-15	3.70E-12
Defense response	2.90E-11	2.40E-08
Secondary metabolite biosynthetic process	1.20E-07	6.80E-05
Defense response to other organisms	2.30E-05	9.40E-03

Supplementary Table S15: Go term enrichment of PAVs for PopY

GO term	P-value	Benjamini-corrected P-value
Signal transduction	1.90E-15	3.20E-12
Defense response	6.60E-15	5.50E-12
Secondary metabolite biosynthetic process	4.10E-08	2.30E-05
Oxidation-reduction process	1.40E-05	5.80E-03
Proteolysis	4.70E-05	1.60E-02
Positive regulation of proteasomal ubiquitin-dependent protein catabolic process	1.40E-04	3.70E-02
Defense response to other organisms	1.40E-04	3.70E-02

Supplementary Table S16: Go term enrichment of PAVs for Riff

GO term	P-value	Benjamini-corrected P-value
Signal transduction	4.80E-14	8.80E-11
Defense response	7.10E-12	6.50E-09
Secondary metabolite biosynthetic process	4.50E-05	2.70E-02
Glucosinolate catabolic process	1.10E-04	4.80E-02

Supplementary Table S17: Go term enrichment of PAVs for South Middle Atlas

GO term	P-value	Benjamini-corrected P-value
Signal transduction	8.40E-19	1.60E-15
Defense response	3.60E-15	3.50E-12
Secondary metabolite biosynthetic process	1.10E-05	7.20E-03
Terpenoid biosynthetic process	2.00E-05	9.70E-03

Supplementary Table S18: Go term enrichment of PAVs for South Sweden

GO term	P-value	Benjamini-corrected P-value
Signal transduction	1.70E-17	4.10E-14
Defense response	1.40E-12	1.70E-09
Secondary metabolite biosynthetic process	8.80E-09	6.90E-06
Flavonoid glucuronidation	3.50E-06	2.00E-03
Terpenoid biosynthetic process	1.20E-05	5.90E-03
Flavonoid biosynthetic process	1.90E-05	7.60E-03
Metabolic process	5.40E-05	1.80E-02
Killing of cells of other organisms	7.20E-05	2.10E-02

Supplementary Table S19: Go term enrichment of PAVs for Western Europe

GO term	P-value	Benjamini-corrected P-value
Signal transduction	1.90E-16	5.40E-13
Defense response	1.30E-14	1.50E-11
Flavonoid biosynthetic process	4.40E-05	3.60E-02
SCF-dependent proteasomal ubiquitin-dependent protein catabolic process	5.20E-05	3.10E-02
Secondary metabolite biosynthetic process	8.80E-05	4.20E-02
Positive regulation of proteasomal ubiquitin-dependent protein catabolic process	1.10E-04	4.40E-02
Regulation of salicylic acid mediated signaling pathway	1.30E-04	4.30E-02
Terpenoid biosynthetic process	1.60E-04	4.80E-02

Supplementary Table S20: Tajima's D calculation for each population including defense, genic and intergenic regions.

Geographic region	Population	Whole genome	Defense genes	All genic	Intergenic	P-Value (defense versus resampled gene sets)
Africa - Morocco	High Atlas	0.236	0.737	0.293	0.211	$1x10^{-5}$
	South Middle Atlas	0.051	0.594	0.111	0.025	$1x10^{-5}$
	North Middle Atlas	0.232	0.537	0.272	0.214	$2.14x10^{-3}$
	Riff	0.093	0.541	0.075	0.102	$1x10^{-5}$
Macaronesia	Madeira	0.152	0.492	0.155	0.150	$7x10^{-4}$
Europe	Iberian Relicts	-0.184	0.409	-0.107	-0.219	$1x10^{-5}$
	Iberian Non-Relicts	-0.222	0.501	-0.087	-0.278	$1x10^{-5}$
	Western Europe	-0.323	0.670	-0.198	-0.372	$1x10^{-5}$
	Central Europe	-0.415	0.636	-0.251	-0.479	$1x10^{-5}$
	Italy, Balkans and Caucasus	-0.447	0.442	-0.281	-0.512	$1x10^{-5}$
	Germany	-0.533	0.190	-0.420	-0.580	$1x10^{-5}$
	South Sweden	0.134	0.993	0.389	0.036	$1x10^{-5}$
	North Sweden	0.492	0.820	0.634	0.433	$2.54x10^{-2}$
Asia	Asia – 1001 Genomes Project samples	-0.121	0.501	-0.052	-0.150	$1x10^{-5}$
	North-Western China (PopN)	0.690	1.181	0.823	0.634	$7.1x10^{-4}$
	Yangtze River Basin (PopY)	0.512	0.545	0.565	0.490	$5.51x10^{-1}$

Supplementary Table S21: Defense response enrichments with betascan results for each population (top 5%)

Geographic region	Number of samples	Beta Score (5%)	Benjamini-corrected P-value
Asia	118	26.15	$1x10^{-3}$
Morocco	61	17.60	$3.3x10^{-3}$
Iberia	139	8.55	$2.2x10^{-4}$
Central Europe	183	11.70	$1.8x10^{-9}$
Germany	156	20.95	$7.7x10^{-6}$
Italy, Balkans, and Caucasus	92	10.98	$4.4x10^{-4}$
North Sweden	64	24.99	$8.1x10^{-5}$
South Sweden	156	14.94	$7.1x10^{-8}$
Western Europe	116	12.89	$1.3x10^{-5}$

Supplementary Table S22: Defense response related PAVs that were identified in 1301 samples

ID	Gene Name
AT1G12010	2-oxoglutarate (2OG) and Fe(II)-dependent oxygenase superfamily protein(AT1G12010)
AT2G19590	ACC oxidase 1(ACO1)
AT1G62380	ACC oxidase 2(ACO2)
AT4G33300	ADR1-like 1(ADR1-L1)
AT4G02150	ARM repeat superfamily protein(MOS6)
AT3G27670	ARM repeat superfamily protein(RST1)
AT1G57650	ATP binding protein(AT1G57650)
AT5G43590	Acyl transferase/acyl hydrolase/lysophospholipase superfamily protein(AT5G43590)
AT2G39030	Acyl-CoA N-acyltransferases (NAT) superfamily protein(NATA1)
AT3G61190	BON association protein 1(BAP1)
AT3G17980	Calcium-dependent lipid-binding (CaLB domain) family protein(C2)
AT1G08860	Calcium-dependent phospholipid-binding Copine family protein(BON3)
AT5G05170	Cellulose synthase family protein(CEV1)
AT2G43600	Chitinase family protein(AT2G43600)
AT2G23960	Class I glutamine amidotransferase-like superfamily protein(AT2G23960)
AT5G15410	Cyclic nucleotide-regulated ion channel family protein(DND1)
AT5G17890	DA1-related protein 4(DAR4)
AT1G33560	Disease resistance protein (CC-NBS-LRR class) family(ADR1)
AT1G53350	Disease resistance protein (CC-NBS-LRR class) family(AT1G53350)
AT1G58390	Disease resistance protein (CC-NBS-LRR class) family(AT1G58390)
AT1G58400	Disease resistance protein (CC-NBS-LRR class) family(AT1G58400)
AT1G58410	Disease resistance protein (CC-NBS-LRR class) family(AT1G58410)
AT1G62630	Disease resistance protein (CC-NBS-LRR class) family(AT1G62630)
AT1G63350	Disease resistance protein (CC-NBS-LRR class) family(AT1G63350)
AT1G63360	Disease resistance protein (CC-NBS-LRR class) family(AT1G63360)
AT5G35450	Disease resistance protein (CC-NBS-LRR class) family(AT5G35450)
AT5G43730	Disease resistance protein (CC-NBS-LRR class) family(AT5G43730)
AT5G43740	Disease resistance protein (CC-NBS-LRR class) family(AT5G43740)
AT5G66900	Disease resistance protein (CC-NBS-LRR class) family(AT5G66900)
AT5G66910	Disease resistance protein (CC-NBS-LRR class) family(AT5G66910)
AT1G59620	Disease resistance protein (CC-NBS-LRR class) family(CW9)
AT5G43470	Disease resistance protein (CC-NBS-LRR class) family(RPP8)
AT1G12220	Disease resistance protein (CC-NBS-LRR class) family(RPS5)
AT5G38350	Disease resistance protein (NBS-LRR class) family(AT5G38350)
AT1G17615	Disease resistance protein (TIR-NBS class)(AT1G17615)
AT1G66090	Disease resistance protein (TIR-NBS class)(AT1G66090)
AT1G72850	Disease resistance protein (TIR-NBS class)(AT1G72850)
AT1G72870	Disease resistance protein (TIR-NBS class)(AT1G72870)
AT1G72890	Disease resistance protein (TIR-NBS class)(AT1G72890)
AT1G72950	Disease resistance protein (TIR-NBS class)(AT1G72950)
AT3G04210	Disease resistance protein (TIR-NBS class)(AT3G04210)
AT1G17600	Disease resistance protein (TIR-NBS-LRR class) family(AT1G17600)
AT1G31540	Disease resistance protein (TIR-NBS-LRR class) family(AT1G31540)
AT1G56520	Disease resistance protein (TIR-NBS-LRR class) family(AT1G56520)
AT1G56540	Disease resistance protein (TIR-NBS-LRR class) family(AT1G56540)
AT1G63730	Disease resistance protein (TIR-NBS-LRR class) family(AT1G63730)
AT1G63740	Disease resistance protein (TIR-NBS-LRR class) family(AT1G63740)
AT1G63750	Disease resistance protein (TIR-NBS-LRR class) family(AT1G63750)
AT1G63870	Disease resistance protein (TIR-NBS-LRR class) family(AT1G63870)
AT1G63880	Disease resistance protein (TIR-NBS-LRR class) family(AT1G63880)
AT1G65850	Disease resistance protein (TIR-NBS-LRR class) family(AT1G65850)

AT2G14080	Disease resistance protein (TIR-NBS-LRR class) family(AT2G14080)
AT2G16870	Disease resistance protein (TIR-NBS-LRR class) family(AT2G16870)
AT2G17060	Disease resistance protein (TIR-NBS-LRR class) family(AT2G17060)
AT3G04220	Disease resistance protein (TIR-NBS-LRR class) family(AT3G04220)
AT3G44400	Disease resistance protein (TIR-NBS-LRR class) family(AT3G44400)
AT3G44630	Disease resistance protein (TIR-NBS-LRR class) family(AT3G44630)
AT3G44670	Disease resistance protein (TIR-NBS-LRR class) family(AT3G44670)
AT3G51560	Disease resistance protein (TIR-NBS-LRR class) family(AT3G51560)
AT3G51570	Disease resistance protein (TIR-NBS-LRR class) family(AT3G51570)
AT4G08450	Disease resistance protein (TIR-NBS-LRR class) family(AT4G08450)
AT4G11170	Disease resistance protein (TIR-NBS-LRR class) family(AT4G11170)
AT4G11340	Disease resistance protein (TIR-NBS-LRR class) family(AT4G11340)
AT4G12010	Disease resistance protein (TIR-NBS-LRR class) family(AT4G12010)
AT4G14370	Disease resistance protein (TIR-NBS-LRR class) family(AT4G14370)
AT4G16900	Disease resistance protein (TIR-NBS-LRR class) family(AT4G16900)
AT4G16920	Disease resistance protein (TIR-NBS-LRR class) family(AT4G16920)
AT4G16940	Disease resistance protein (TIR-NBS-LRR class) family(AT4G16940)
AT4G23510	Disease resistance protein (TIR-NBS-LRR class) family(AT4G23510)
AT4G36150	Disease resistance protein (TIR-NBS-LRR class) family(AT4G36150)
AT5G17970	Disease resistance protein (TIR-NBS-LRR class) family(AT5G17970)
AT5G18350	Disease resistance protein (TIR-NBS-LRR class) family(AT5G18350)
AT5G18360	Disease resistance protein (TIR-NBS-LRR class) family(AT5G18360)
AT5G22690	Disease resistance protein (TIR-NBS-LRR class) family(AT5G22690)
AT5G36930	Disease resistance protein (TIR-NBS-LRR class) family(AT5G36930)
AT5G38340	Disease resistance protein (TIR-NBS-LRR class) family(AT5G38340)
AT5G40100	Disease resistance protein (TIR-NBS-LRR class) family(AT5G40100)
AT5G40910	Disease resistance protein (TIR-NBS-LRR class) family(AT5G40910)
AT5G41540	Disease resistance protein (TIR-NBS-LRR class) family(AT5G41540)
AT5G41550	Disease resistance protein (TIR-NBS-LRR class) family(AT5G41550)
AT5G41740	Disease resistance protein (TIR-NBS-LRR class) family(AT5G41740)
AT5G41750	Disease resistance protein (TIR-NBS-LRR class) family(AT5G41750)
AT5G45000	Disease resistance protein (TIR-NBS-LRR class) family(AT5G45000)
AT5G45060	Disease resistance protein (TIR-NBS-LRR class) family(AT5G45060)
AT5G45200	Disease resistance protein (TIR-NBS-LRR class) family(AT5G45200)
AT5G45210	Disease resistance protein (TIR-NBS-LRR class) family(AT5G45210)
AT5G45220	Disease resistance protein (TIR-NBS-LRR class) family(AT5G45220)
AT5G45230	Disease resistance protein (TIR-NBS-LRR class) family(AT5G45230)
AT5G46270	Disease resistance protein (TIR-NBS-LRR class) family(AT5G46270)
AT5G46450	Disease resistance protein (TIR-NBS-LRR class) family(AT5G46450)
AT5G46490	Disease resistance protein (TIR-NBS-LRR class) family(AT5G46490)
AT5G46510	Disease resistance protein (TIR-NBS-LRR class) family(AT5G46510)
AT5G46520	Disease resistance protein (TIR-NBS-LRR class) family(AT5G46520)
AT5G48770	Disease resistance protein (TIR-NBS-LRR class) family(AT5G48770)
AT5G49140	Disease resistance protein (TIR-NBS-LRR class) family(AT5G49140)
AT5G51630	Disease resistance protein (TIR-NBS-LRR class) family(AT5G51630)
AT5G58120	Disease resistance protein (TIR-NBS-LRR class) family(AT5G58120)
AT5G44870	Disease resistance protein (TIR-NBS-LRR class) family(LAZ5)
AT1G64070	Disease resistance protein (TIR-NBS-LRR class) family(RLM1)
AT3G44480	Disease resistance protein (TIR-NBS-LRR class) family(RPP1)
AT4G16860	Disease resistance protein (TIR-NBS-LRR class) family(RPP4)
AT4G16950	Disease resistance protein (TIR-NBS-LRR class) family(RPP5)
AT1G72840	Disease resistance protein (TIR-NBS-LRR class)(AT1G72840)
AT4G19510	Disease resistance protein (TIR-NBS-LRR class)(AT4G19510)

AT5G11250	Disease resistance protein (TIR-NBS-LRR class)(AT5G11250)
AT5G38850	Disease resistance protein (TIR-NBS-LRR class)(AT5G38850)
AT5G45240	Disease resistance protein (TIR-NBS-LRR class)(AT5G45240)
AT5G45260	Disease resistance protein (TIR-NBS-LRR class)(RRS1)
AT5G45050	Disease resistance protein (TIR-NBS-LRR class)(TTR1)
AT1G56510	Disease resistance protein (TIR-NBS-LRR class)(WRR4)
AT1G22900	Disease resistance-responsive (dirigent-like protein) family protein(AT1G22900)
AT1G58170	Disease resistance-responsive (dirigent-like protein) family protein(AT1G58170)
AT1G65870	Disease resistance-responsive (dirigent-like protein) family protein(AT1G65870)
AT2G21100	Disease resistance-responsive (dirigent-like protein) family protein(AT2G21100)
AT3G13660	Disease resistance-responsive (dirigent-like protein) family protein(AT3G13660)
AT3G13662	Disease resistance-responsive (dirigent-like protein) family protein(AT3G13662)
AT3G55230	Disease resistance-responsive (dirigent-like protein) family protein(AT3G55230)
AT4G11180	Disease resistance-responsive (dirigent-like protein) family protein(AT4G11180)
AT4G11190	Disease resistance-responsive (dirigent-like protein) family protein(AT4G11190)
AT4G11210	Disease resistance-responsive (dirigent-like protein) family protein(AT4G11210)
AT5G42500	Disease resistance-responsive (dirigent-like protein) family protein(AT5G42500)
AT5G42510	Disease resistance-responsive (dirigent-like protein) family protein(AT5G42510)
AT5G49040	Disease resistance-responsive (dirigent-like protein) family protein(AT5G49040)
AT1G64160	Disease resistance-responsive (dirigent-like protein) family protein(DIR5)
AT1G31580	ECS1(ECS1)
AT2G17310	F-box and associated interaction domains-containing protein(SON1)
AT3G62980	F-box/RNI-like superfamily protein(TIR1)
AT2G43910	HARMLESS TO OZONE LAYER 1(HOL1)
AT3G50950	HOPZ-ACTIVATED RESISTANCE 1(ZAR1)
AT1G19100	Histidine kinase-, DNA gyrase B-, and HSP90-like ATPase family protein(DMS11)
AT3G09710	IQ-domain 1(IQD1)
AT2G40220	Integrase-type DNA-binding superfamily protein(ABI4)
AT1G58602	LRR and NB-ARC domains-containing disease resistance protein(AT1G58602)
AT1G61180	LRR and NB-ARC domains-containing disease resistance protein(AT1G61180)
AT1G61190	LRR and NB-ARC domains-containing disease resistance protein(AT1G61190)
AT1G61300	LRR and NB-ARC domains-containing disease resistance protein(AT1G61300)
AT1G61310	LRR and NB-ARC domains-containing disease resistance protein(AT1G61310)
AT3G14460	LRR and NB-ARC domains-containing disease resistance protein(AT3G14460)
AT4G10780	LRR and NB-ARC domains-containing disease resistance protein(AT4G10780)
AT5G05400	LRR and NB-ARC domains-containing disease resistance protein(AT5G05400)
AT5G47250	LRR and NB-ARC domains-containing disease resistance protein(AT5G47250)
AT1G12280	LRR and NB-ARC domains-containing disease resistance protein(SUMM2)
AT3G44220	Late embryogenesis abundant (LEA) hydroxyproline-rich glycoprotein family(AT3G44220)
AT2G26380	Leucine-rich repeat (LRR) family protein(AT2G26380)
AT3G20820	Leucine-rich repeat (LRR) family protein(AT3G20820)
AT2G31880	Leucine-rich repeat protein kinase family protein(SOBIR1)
AT2G18170	MAP kinase 7(MPK7)
AT5G23820	MD-2-related lipid recognition domain-containing protein(AT5G23820)
AT1G35260	MLP-like protein 165(MLP165)
AT1G35310	MLP-like protein 168(MLP168)
AT1G70830	MLP-like protein 28(MLP28)
AT1G70840	MLP-like protein 31(MLP31)
AT2G01520	MLP-like protein 328(MLP328)
AT1G24020	MLP-like protein 423(MLP423)
AT1G70890	MLP-like protein 43(MLP43)
AT3G61220	NAD(P)-binding Rossmann-fold superfamily protein(SDR1)

AT5G51060	NADPH/respiratory burst oxidase protein D(RHD2)
AT1G50180	NB-ARC domain-containing disease resistance protein(AT1G50180)
AT1G59780	NB-ARC domain-containing disease resistance protein(AT1G59780)
AT3G14470	NB-ARC domain-containing disease resistance protein(AT3G14470)
AT3G46710	NB-ARC domain-containing disease resistance protein(AT3G46710)
AT3G46730	NB-ARC domain-containing disease resistance protein(AT3G46730)
AT4G09360	NB-ARC domain-containing disease resistance protein(AT4G09360)
AT4G27190	NB-ARC domain-containing disease resistance protein(AT4G27190)
AT4G27220	NB-ARC domain-containing disease resistance protein(AT4G27220)
AT1G10920	NB-ARC domain-containing disease resistance protein(LOV1)
AT3G07040	NB-ARC domain-containing disease resistance protein(RPM1)
AT3G46530	NB-ARC domain-containing disease resistance protein(RPP13)
AT4G09590	NDR1/HIN1-like 22(NHL22)
AT1G60800	NSP-interacting kinase 3(NIK3)
AT1G66250	O-Glycosyl hydrolases family 17 protein(AT1G66250)
AT2G01630	O-Glycosyl hydrolases family 17 protein(AT2G01630)
AT4G31140	O-Glycosyl hydrolases family 17 protein(AT4G31140)
AT5G58090	O-Glycosyl hydrolases family 17 protein(AT5G58090)
AT4G14080	O-Glycosyl hydrolases family 17 protein(MEE48)
AT4G19060	P-loop containing nucleoside triphosphate hydrolases superfamily protein(AT4G19060)
AT5G45440	P-loop containing nucleoside triphosphate hydrolases superfamily protein(AT5G45440)
AT5G45490	P-loop containing nucleoside triphosphate hydrolases superfamily protein(AT5G45490)
AT4G37050	PATATIN-like protein 4(PLP4)
AT2G39220	PATATIN-like protein 6(PLP6)
AT5G55240	PEROXYGENASE 2(ATPXG2)
AT2G37040	PHE ammonia lyase 1(PAL1)
AT2G31980	PHYTOCYSTATIN 2(CYS2)
AT1G77630	Peptidoglycan-binding LysM domain-containing protein(LYM3)
AT2G15130	Plant basic secretory protein (BSP) family protein(AT2G15130)
AT2G43710	Plant stearoyl-acyl-carrier-protein desaturase family protein(SSI2)
AT5G45560	Pleckstrin homology (PH) domain-containing protein / lipid-binding START domain-containing protein(AT5G45560)
AT5G58600	Pmr5/Cas1p GDSL/SGNH-like acyl-esterase family protein (DUF828)(PMR5)
AT1G14930	Polyketide cyclase/dehydrase and lipid transport superfamily protein(AT1G14930)
AT1G24000	Polyketide cyclase/dehydrase and lipid transport superfamily protein(AT1G24000)
AT1G30990	Polyketide cyclase/dehydrase and lipid transport superfamily protein(AT1G30990)
AT3G26450	Polyketide cyclase/dehydrase and lipid transport superfamily protein(AT3G26450)
AT3G26460	Polyketide cyclase/dehydrase and lipid transport superfamily protein(AT3G26460)
AT4G14060	Polyketide cyclase/dehydrase and lipid transport superfamily protein(AT4G14060)
AT4G23680	Polyketide cyclase/dehydrase and lipid transport superfamily protein(AT4G23680)
AT5G28000	Polyketide cyclase/dehydrase and lipid transport superfamily protein(AT5G28000)
AT1G55870	Polynucleotidyl transferase, ribonuclease H-like superfamily protein(AHG2)
AT4G11890	Protein kinase superfamily protein(ARCK1)
AT2G30740	Protein kinase superfamily protein(AT2G30740)
AT4G28910	Putative interactor of JAZ(NINJA)
AT3G03450	RGA-like 2(RGL2)
AT4G35480	RING-H2 finger A3B(RHA3B)
AT4G15975	RING/U-box superfamily protein(AT4G15975)
AT2G41060	RNA-binding (RRM/RBD/RNP motifs) family protein(AT2G41060)
AT1G12210	RPS5-like 1(RFL1)
AT3G11480	S-adenosyl-L-methionine-dependent methyltransferases superfamily protein(BSMT1)
AT1G80680	SUPPRESSOR OF AUXIN RESISTANCE 3(SAR3)
AT2G02120	Scorpion toxin-like knottin superfamily protein(PDF2.1)

AT2G38900	Serine protease inhibitor, potato inhibitor I-type family protein(AT2G38900)
AT3G50020	Serine protease inhibitor, potato inhibitor I-type family protein(AT3G50020)
AT5G43570	Serine protease inhibitor, potato inhibitor I-type family protein(AT5G43570)
AT4G02600	Seven transmembrane MLO family protein(MLO1)
AT5G65970	Seven transmembrane MLO family protein(MLO10)
AT4G24250	Seven transmembrane MLO family protein(MLO13)
AT1G26700	Seven transmembrane MLO family protein(MLO14)
AT1G11310	Seven transmembrane MLO family protein(MLO2)
AT3G45290	Seven transmembrane MLO family protein(MLO3)
AT2G33670	Seven transmembrane MLO family protein(MLO5)
AT1G61560	Seven transmembrane MLO family protein(MLO6)
AT1G42560	Seven transmembrane MLO family protein(MLO9)
AT1G66340	Signal transduction histidine kinase, hybrid-type, ethylene sensor(ETR1)
AT1G74950	TIFY domain/Divergent CCT motif family protein(TIFY10B)
AT4G16890	TIR-NBS-LRR class disease resistance protein(SNC1)
AT3G16720	TOXICOS EN LEVADURA 2(ATL2)
AT3G06730	Thioredoxin z(TRX z)
AT1G47370	Toll-Interleukin-Resistance (TIR) domain family protein(AT1G47370)
AT1G57630	Toll-Interleukin-Resistance (TIR) domain family protein(AT1G57630)
AT1G57670	Toll-Interleukin-Resistance (TIR) domain family protein(AT1G57670)
AT1G57850	Toll-Interleukin-Resistance (TIR) domain family protein(AT1G57850)
AT1G60320	Toll-Interleukin-Resistance (TIR) domain family protein(AT1G60320)
AT1G72920	Toll-Interleukin-Resistance (TIR) domain family protein(AT1G72920)
AT2G03030	Toll-Interleukin-Resistance (TIR) domain family protein(AT2G03030)
AT2G03300	Toll-Interleukin-Resistance (TIR) domain family protein(AT2G03300)
AT4G04110	Toll-Interleukin-Resistance (TIR) domain family protein(AT4G04110)
AT4G19910	Toll-Interleukin-Resistance (TIR) domain family protein(AT4G19910)
AT4G19920	Toll-Interleukin-Resistance (TIR) domain family protein(AT4G19920)
AT4G19925	Toll-Interleukin-Resistance (TIR) domain family protein(AT4G19925)
AT4G23515	Toll-Interleukin-Resistance (TIR) domain family protein(AT4G23515)
AT5G44900	Toll-Interleukin-Resistance (TIR) domain family protein(AT5G44900)
AT5G44920	Toll-Interleukin-Resistance (TIR) domain family protein(AT5G44920)
AT1G72900	Toll-Interleukin-Resistance (TIR) domain-containing protein(AT1G72900)
AT1G72910	Toll-Interleukin-Resistance (TIR) domain-containing protein(AT1G72910)
AT1G72940	Toll-Interleukin-Resistance (TIR) domain-containing protein(AT1G72940)
AT4G16930	Toll-Interleukin-Resistance (TIR) domain-containing protein(AT4G16930)
AT3G56860	UBP1-associated protein 2A(UBA2A)
AT4G34131	UDP-glucosyl transferase 73B3(UGT73B3)
AT2G15480	UDP-glucosyl transferase 73B5(UGT73B5)
AT3G57860	UV-B-insensitive 4-like protein(UVI4-LIKE)
AT1G43700	VIRE2-interacting protein 1(VIP1)
AT1G32585	VQ motif-containing protein-like protein(AT1G32585)
AT1G79680	WALL ASSOCIATED KINASE (WAK)-LIKE 10(WAKL10)
AT5G15130	WRKY DNA-binding protein 72(WRKY72)
AT4G01250	WRKY family transcription factor(WRKY22)
AT3G48080	alpha/beta-Hydrolases superfamily protein(AT3G48080)
AT3G48090	alpha/beta-Hydrolases superfamily protein(EDS1)
AT1G65880	benzoyloxyglucosinolate 1(BZO1)
AT3G57270	beta-1,3-glucanase 1(BG1)
AT5G20330	beta-1,3-glucanase 4(BETAG4)
AT5G20340	beta-1,3-glucanase 5(BG5)
AT5G42100	beta-1,3-glucanase(BG_PPAP)
AT4G08920	cryptochrome 1(CRY1)

AT2G46450	cyclic nucleotide-gated channel 12(CNGC12)
AT2G46440	cyclic nucleotide-gated channels(CNGC11)
AT5G51700	cysteine and histidine-rich domain-containing protein RAR1(PBS2)
AT4G23280	cysteine-rich RLK (RECEPTOR-like protein kinase) 20(CRK20)
AT4G23310	cysteine-rich RLK (RECEPTOR-like protein kinase) 23(CRK23)
AT3G45860	cysteine-rich RLK (RECEPTOR-like protein kinase) 4(CRK4)
AT2G30770	cytochrome P450 family 71 polypeptide(CYP71A13)
AT4G39950	cytochrome P450, family 79, subfamily B, polypeptide 2(CYP79B2)
AT3G25180	cytochrome P450, family 82, subfamily G, polypeptide 1(CYP82G1)
AT1G19570	dehydroascorbate reductase(DHAR1)
AT5G57690	diacylglycerol kinase 4(DGK4)
AT5G07920	diacylglycerol kinase1(DGK1)
AT1G51480	disease resistance protein (CC-NBS-LRR class) family protein(AT1G51480)
AT5G48780	disease resistance protein (TIR-NBS class)(AT5G48780)
AT4G16990	disease resistance protein (TIR-NBS class)(RLM3)
AT3G25510	disease resistance protein (TIR-NBS-LRR class) family protein(AT3G25510)
AT5G18370	disease resistance protein (TIR-NBS-LRR class) family protein(AT5G18370)
AT4G19520	disease resistance protein (TIR-NBS-LRR class) family(AT4G19520)
AT4G19530	disease resistance protein (TIR-NBS-LRR class) family(AT4G19530)
AT5G46260	disease resistance protein (TIR-NBS-LRR class) family(AT5G46260)
AT5G46470	disease resistance protein (TIR-NBS-LRR class) family(RPS6)
AT1G27180	disease resistance protein (TIR-NBS-LRR class)(AT1G27180)
AT1G69550	disease resistance protein (TIR-NBS-LRR class)(AT1G69550)
AT2G17050	disease resistance protein (TIR-NBS-LRR class)(AT2G17050)
AT4G09430	disease resistance protein (TIR-NBS-LRR class)(AT4G09430)
AT4G16960	disease resistance protein (TIR-NBS-LRR class)(AT4G16960)
AT4G36140	disease resistance protein (TIR-NBS-LRR class)(AT4G36140)
AT5G17680	disease resistance protein (TIR-NBS-LRR class)(AT5G17680)
AT5G17880	disease resistance protein (TIR-NBS-LRR class)(CSA1)
AT5G44210	erf domain protein 9(ERF9)
AT3G23240	ethylene response factor 1(ERF1)
AT3G20310	ethylene response factor 7(ERF7)
AT1G05010	ethylene-forming enzyme(EFE)
AT4G03550	glucan synthase-like 5(GSL05)
AT2G47730	glutathione S-transferase phi 8(GSTF8)
AT5G56030	heat shock protein 81-2(HSP81-2)
AT1G18870	isochorismate synthase 2(ICS2)
AT3G43440	jasmonate-zim-domain protein 11(JAZ11)
AT5G20900	jasmonate-zim-domain protein 12(JAZ12)
AT3G17860	jasmonate-zim-domain protein 3(JAZ3)
AT1G48500	jasmonate-zim-domain protein 4(JAZ4)
AT1G17380	jasmonate-zim-domain protein 5(JAZ5)
AT2G34600	jasmonate-zim-domain protein 7(JAZ7)
AT1G30135	jasmonate-zim-domain protein 8(JAZ8)
AT1G55020	lipoxygenase 1(LOX1)
AT2G02130	low-molecular-weight cysteine-rich 68(LCR68)
AT2G02100	low-molecular-weight cysteine-rich 69(LCR69)
AT2G02140	low-molecular-weight cysteine-rich 72(LCR72)
AT5G38330	low-molecular-weight cysteine-rich 80(LCR80)
AT2G17120	lysm domain GPI-anchored protein 2 precursor(LYM2)
AT1G02170	metacaspase 1(MC1)
AT3G55270	mitogen-activated protein kinase phosphatase 1(MKP1)
AT3G06490	myb domain protein 108(MYB108)

AT1G52040	myrosinase-binding protein 1(MBP1)
AT1G52030	myrosinase-binding protein 2(MBP2)
AT4G19500	nucleoside-triphosphatase/transmembrane receptor/nucleotide binding/ATP binding protein(AT4G19500)
AT3G22231	pathogen and circadian controlled 1(PCC1)
AT4G29940	pathogenesis related homeodomain protein A(PRHA)
AT2G14610	pathogenesis-related protein 1(PR1)
AT3G49110	peroxidase CA(PRXCA)
AT3G49120	peroxidase CB(PRXCB)
AT5G04230	phenyl alanine ammonia-lyase 3(PAL3)
AT3G53260	phenylalanine ammonia-lyase 2(PAL2)
AT1G65390	phloem protein 2 A5(PP2-A5)
AT5G45090	phloem protein 2-A7(PP2-A7)
AT4G23570	phosphatase-like protein(SGT1A)
AT5G44420	plant defensin 1.2(PDF1.2)
AT5G44430	plant defensin 1.2C(PDF1.2c)
AT2G26020	plant defensin 1.2b(PDF1.2b)
AT2G26010	plant defensin 1.3(PDF1.3)
AT1G55010	plant defensin 1.5(PDF1.5)
AT5G06860	polygalacturonase inhibiting protein 1(PGIP1)
AT5G06870	polygalacturonase inhibiting protein 2(PGIP2)
AT4G12020	protein kinase family protein(WRKY19)
AT5G47260	putative disease resistance protein(AT5G47260)
AT1G71390	receptor like protein 11(RLP11)
AT2G15080	receptor like protein 19(RLP19)
AT2G33050	receptor like protein 26(RLP26)
AT2G33060	receptor like protein 27(RLP27)
AT3G05360	receptor like protein 30(RLP30)
AT3G05370	receptor like protein 31(RLP31)
AT3G05650	receptor like protein 32(RLP32)
AT3G05660	receptor like protein 33(RLP33)
AT3G11010	receptor like protein 34(RLP34)
AT3G11080	receptor like protein 35(RLP35)
AT3G23010	receptor like protein 36(RLP36)
AT3G23110	receptor like protein 37(RLP37)
AT3G23120	receptor like protein 38(RLP38)
AT3G24900	receptor like protein 39(RLP39)
AT3G25010	receptor like protein 41(RLP41)
AT3G25020	receptor like protein 42(RLP42)
AT4G13810	receptor like protein 47(RLP47)
AT4G13920	receptor like protein 50(RLP50)
AT5G25910	receptor like protein 52(RLP52)
AT5G27060	receptor like protein 53(RLP53)
AT1G45616	receptor like protein 6(RLP6)
AT1G47890	receptor like protein 7(RLP7)
AT5G07390	respiratory burst oxidase homolog A(RBOHA)
AT4G02380	senescence-associated gene 21(SAG21)
AT3G15390	silencing defective 5(SDE5)
AT1G14410	ssDNA-binding transcriptional regulator(WHY1)
AT1G66980	suppressor of npr1-1 constitutive 4(SNC4)
AT5G44510	target of AVRB operation1(TAO1)
AT1G72260	thionin 2.1(THI2.1)
AT5G36910	thionin 2.2(THI2.2)

AT1G12660	thionin-like protein(AT1G12660)
AT1G12663	thionin-like protein(AT1G12663)
AT1G72930	toll/interleukin-1 receptor-like protein(TIR)
AT2G32140	transmembrane receptor(AT2G32140)
AT1G27170	transmembrane receptors / ATP binding protein(AT1G27170)
AT2G43520	trypsin inhibitor protein 2(TI2)
AT2G42260	uv-b-insensitive 4(UVI4)
AT5G24780	vegetative storage protein 1(VSP1)
